# SPOCK1, as a potential prognostic and therapeutic biomarker for lung adenocarcinoma, is associated with epithelial-mesenchymal transition and immune evasion

**DOI:** 10.1186/s12967-023-04616-3

**Published:** 2023-12-12

**Authors:** Yafeng Liu, Tao Han, Jing Wu, Jiawei Zhou, Jianqiang Guo, Rui Miao, Zhi Xu, Yingru Xing, Ying Bai, Dong Hu

**Affiliations:** 1 School of Medicine, Anhui University of Science and Technology, Chongren Building, No 168, Taifeng St, Huainan, 232001 People’s Republic of China; 2 Anhui Province Engineering Laboratory of Occupational Health and Safety, Anhui University of Science and Technology, Huainan, People’s Republic of China; 3 Key Laboratory of Industrial Dust Prevention and Control & Occupational Safety and Health of the Ministry of Education, Anhui University of Science and Technology, Huainan, People’s Republic of China; 4 Department of Clinical Laboratory, Anhui Zhongke Gengjiu Hospital, Hefei, People’s Republic of China; 5 Department of Laboratory Medicine, The First Affiliated Hospital of USTC, Division of Life Sciences and Medicine, University of Science and Technology of China, Hefei, China

**Keywords:** Lung adenocarcinoma, Epithelial-mesenchymal transition, Immune evasion, TIDE, SPOCK1

## Abstract

**Background:**

The occurrence of epithelial-mesenchymal transition (EMT) and immune evasion is considered to contribute to poor prognosis in lung adenocarcinoma (LUAD). Therefore, this study aims to explore the key oncogenes that promote EMT and immune evasion and reveal the expression patterns, prognostic value, and potential biological functions.

**Methods:**

Firstly, we identified gene modules associated with EMT and Tumor Immune Dysfunction and Exclusion (TIDE) through weighted gene co-expression network analysis (WGCNA). Next, we utilized differential analysis and machine learning to identify the key genes and validate them. Moreover, we analyzed the correlation between key genes and tumor microenvironment remodeling, drug sensitivity, as well as mutation frequency. Furthermore, we explored and validated their malignant biological characteristics through in vitro experiments and clinical samples. Finally, potential drugs for LUAD were screened based on CMap and validated through experiments.

**Results:**

Firstly, WGCNA analysis revealed that red and green modules were highly correlated with EMT and TIDE. Among them, upregulated expression of SPOCK1 was observed in lung adenocarcinoma tissues and was associated with poor prognosis. Additionally, patients in the high SPOCK1 group showed more activation of malignant oncogenic pathways, higher infiltration of immunosuppressive components, and a higher frequency of mutations. The knockdown of SPOCK1 suppressed invasion and metastasis capabilities of lung adenocarcinoma cells, and the high expression of SPOCK1 was associated with low infiltration of CD8^+^ T cells. Therapeutic aspects, SPOCK1 can be a candidate indicator for drug sensitivity and CMap showed that VER-155008 was the drug candidate with the largest perturbation effect on the SPOCK1 expression profile. In vitro and in vivo experiments validated the cancer-inhibitory effect of VER-155008 in LUAD.

**Conclusion:**

This study revealed through comprehensive bioinformatics analysis and experimental analysis that SPOCK1 can promote EMT and immune escape in LUAD, and it may serve as a promising candidate prognostic biomarker and therapeutic target for LUAD.

**Supplementary Information:**

The online version contains supplementary material available at 10.1186/s12967-023-04616-3.

## Introduction

Lung cancer represents one of the most fatal malignancies globally, with lung adenocarcinoma (LUAD) accounting for 50% of all lung cancer cases [1]. LUAD exhibits a high propensity for metastasis and recurrence, as well as a predisposition to develop resistance toward conventional chemotherapy [2]. Despite substantial therapeutic advancements in recent years, LUAD patients continue to experience disheartening long-term survival rates, with a 5 year survival rate below 20% [3]. Hence, comprehending the etiology and mechanisms driving malignant progression of LUAD is of paramount importance in order to elucidate more efficacious therapeutic approaches.

The ever-growing evidence indicates the role of tumor microenvironment (TME) heterogeneity in impacting cancer patients’ response to anti-cancer therapies [4, 5]. Comprising a range of infiltrating cells, including immune cells, endothelial cells, fibroblasts, and tumor cells, the TME’s diverse components have been associated with divergent clinical outcomes [6]. Infiltration of cytolytic immune cells, particularly CD8^+^ T cells, has emerged as a predictive factor for favorable prognosis in various malignancies due to their role in inducing cancer cell death [7]. Nevertheless, sustained chronic stimulation of cancer-related antigens within the TME or augmented infiltration of anti-inflammatory M2-like macrophages and cancer-associated fibroblasts often impedes the ability of CD8^+^ T cells to induce tumor regression in advanced cancer patients [8]. This phenomenon is primarily driven by disparities in CD8^+^ T cell quantity and functional attributes, compromised antigen presentation processes, and enrichment of immune checkpoint expressions, encapsulating a series of immune evasion mechanisms. Therefore, there is still a need for prognostic biomarkers capable of assessing the intra-tumoral TME profile as well as the level of CD8^+^ T cell function and infiltration to facilitate the precise treatment of LUAD.

EMT is the process of cellular transformation from epithelial to mesenchymal form, which has an important role in the biological processes of development and wound healing [9, 10]. Furthermore, EMT is the key transitional stage during tumor metastasis and represents the most invasive subtype of malignant epithelial tumors. It is associated with various malignant features of tumors, including tumor initiation, cell migration, vascular infiltration, metastasis, and resistance to treatment [11–13]. In addition, successful EMT is also thought to be a common phenotype shared by circulating tumor cells and disseminated tumor cells [14–16]. Thus, EMT progression is often associated with poor prognostic clinical outcomes for patients.

In this study, our aim was to explore potential biological markers associated with immune evasion and EMT progression in lung adenocarcinoma. SPOCK1 (The SPARC/osteonectin, CWCV and Kazal-like domains proteoglycan 1) was identified as a key gene. SPOCK1 is considered a resident of the extracellular matrix [17], which inhibits the apoptosis of tumor cells and promotes EMT formation by participating in the activation of the TGF-β pathway [18]. In addition, overexpression of SPOCK1 in ovarian cancer cells has also been shown to significantly promote the migration level of tumor cells [19]. Our study reveals that SPOCK1 is a poor prognostic marker for lung adenocarcinoma and correlates with invasive metastasis of tumor cells, immunosuppressive tumor microenvironment formation, and low infiltration of CD8^+^ T cells in the tumor region. Therefore, SPOCK1 may serve as a novel therapeutic target and potential immunotherapy and prognostic biomarker for lung adenocarcinoma.

## Materials and methods

### Data download and preprocessing

First, we downloaded the mRNA expression levels and clinical information of LUAD patients from The Cancer Genome Atlas (TCGA) through the UCSC Xena database (https://xenabrowser.net/datapages/). (576 samples, 517 tumor samples and 59 normal samples). After excluding patients without survival time, a total of 502 LUAD patients were included in the follow-up analysis. The GSE72094, GSE13213, GSE31210, GSE68465, and GSE26939 datasets and one early-stage lung adenocarcinoma dataset GSE166722 (containing clinical information on the aggressive phenotype of the patients) were obtained in the Gene Expression Omnibus (GEO) database. Among them, we merged TCGA-LUAD with GSE72094, GSE13213, and GSE31210 datasets (Samples with no survival time were excluded), using the “Combat” method within the “sva” R package to remove batch effects. In addition, we downloaded mutation data from the TCGA database for LUAD patients. Transcriptome expression profiles of non-small cell lung cancer (NSCLC) cell lines were downloaded from Cancer Cell Line Encyclopedia (CCLE) [20].

### Identification of co-expressed gene modules associated with T cell function and EMT pathways

Tumor Immune Dysfunction and Exclusion (TIDE) can be used to evaluate tumor immune evasion. We uploaded the scaled transcriptome expression profiles and calculated the TIDE scores for each patient with lung adenocarcinoma (LUAD) to represent the degree of immune escape within the tumor microenvironment (http://tide.dfci.harvard.edu/). Next, we obtained the EMT signature from the hallmark gene sets in the Molecular Signatures Database (MSigDB, http://www.gseamsigdb.org/gsea/msigdb/index.jsp) and quantified the EMT scores for each LUAD patient using single-sample gene set enrichment analysis (ssGSEA). The ‘WGCNA’ package [21] in R software was performed to construct a co-expression network of the top 75% variant genes, which were computed using a robust method called median absolute deviation (MAD). First, we calculated and chose β = 3 as an appropriate soft threshold β in this study. Afterward, the cutreeDynamic function was used to identify co-expression gene modules with a minimum module size of 30 and a merge height cut of 0.25. The moduleEigengenes (MEs) function in the “WGCNA” package was used to evaluate the variability of MEs. The degree of association between MEs and TIDE score and EMT score in lung adenocarcinoma was assessed.

### Functional enrichment analysis

Functional enrichment analysis was performed using Metascape (http://metascape.org) [22]. GSEA enrichment analysis was performed by identifying biological changes associated with target genes through the “clusterProfiler” R package, with gene sets as hallmark gene sets and alignment set to 10,000 to obtain normalized enrichment scores (NESs). Gene sets adjusted for P values < 0.05 were considered significantly enriched.

### Identification of hub gene based on machine learning approaches

In order to screen out the key genes in the target modules. We used the last absolute shrinkage and selection operator (LASSO) method and random survival forest (RSF) method to obtain overall survival (OS)-associated hub genes, respectively (P < 0.05). We later overlapped the prognostic key genes generated by the two machine learning methods to obtain robust prognostic key genes.

### DNA methylation changes and somatic variations of SPOCK1

We used the UALCAN database (https://ualcan.path.uab.edu/index.html) for the analysis of methylation level changes between normal and tumor and between patients with TP53 mutation and TP53 wild type. The cBioPortal database (https://www.cbioportal.org/) was used to analyze the mutation profile of SPOCK1 gene. In addition, we analyzed the landscape of the top 20 mutated genes ranked by mutation frequency within the high and low SPOCK1 expression groups, using the median SPOCK1 expression as a cut-off.

### Invasive characterization of SPOCK1

The expression distribution of SPOCK1 in the invasive and non-invasive subtypes was analyzed using the GSE166722 dataset [23]. The list of EMT-related marker genes from the study by Shi et al. was obtained and their correlation with SPOCK1 was analyzed [24]. The PROGENy algorithm was employed to evaluate the activation levels of oncogenic pathways in patients [25].

### Western blotting

Treated cells were lysed in RIPA buffer mixed with 1 × PMSF (100 mM). The denatured isolated proteins were separated using a concentrated gel and 10% isolation gel, and the target protein bands were blocked in 5% skim milk at room temperature for 1 h. All membranes were incubated overnight at 4 °C with specific primary antibodies and incubated with secondary antibodies at room temperature for 1 h. Then incubated with ECL for 30 s. The results were measured using an imager.

### Cell culture, transient transfection and reagents

Human lung adenocarcinoma cell lines BEAS-2B, H1299, A549 and H1975; as well as mouse Lewis lung cancer cells were obtained from ATCC and cultured in DMEM high glycemic medium (DMEM; Gibco) containing 10% fetal bovine serum (FBS; Lonsera) and 1% penicillin/streptomycin (Beyotime; C0222). Cells were cultured in a constant temperature incubator (H&ERAcell 150i; Thermo Scientific) at 37 °C in 5% CO2. Lipofectamine 2000 (Invitrogen, USA) was used to transfect Negative Control (NC) and SPOCK1 siRNAs (GenePharma, China) into LUAD cells according to the manufacturer’s instruction. The sequences of the SPOCK1 siRNA were as follows: 5′ GACGAUGAUUAUUUCAGAATT3′ (si-SPOCK1_1); 5′ GACCUUCGAAUUUGGUCAATT3′ (si-SPOCK1_2); and 5′ CUUGCCAGAAUGAAAUGAATT3′ (si-SPOCK1_3). Primary antibodies used were as follows: E-Cadherin (cell signaling technology, 14472S, Mouse mAb), N-Cadherin (cell signaling technology, 13116 T, Rabbit mAb), Vimentin (cell signaling technology, 5741 T, Rabbit mAb), MMP-9 (cell signaling technology, 13667 T, Rabbit mAb), β-Actin (Abclonal, AC026, Rabbit mAb), SPOCK1 (R&D, MAB2327-SP, Mouse mAb). Secondary HRP-conjugated anti-rabbit and anti-mouse antibodies include HRP Goat Anti-Rabbit lgG (H + L) (Abclonal, AS014) and HRP-conjugated Affinipure Goat Anti-Mouse IgG (H + L) (SA00001-1, Proteintech). The drug VER-155008 and Paclitaxel were purchased from Yuanye Company.

### Wound healing

Cells were inoculated in 6-well plates and then SPOCK1 knockdown was performed. When the cells grew to 100% density, the cells were scratched with the 200 μl pipette tip. Images were acquired at 0 or 48 h after scraping and the wound healing rate was analyzed using Image J software.

### Transwell assay

For migration assay, 5 × 10^4^ cells in 200 μl of serum-free DMEM are loaded into the upper chamber of the Transwell chamber. Then, 700 μl of DMEM supplemented with 10% FBS was loaded into the lower chamber. At 48 h after treatment, cells below the membrane were stained with 0.1% crystal violet and photographed under a microscope. Invasion experiments were performed in much the same way as migration, except that in invasion experiments, Transwell chambers needed to be wrapped with matrix gel before performing the experiment.

### Exploration of immune infiltration in the tumor microenvironment

We obtained 29 gene expression features from the study by Bagaev et al. that describe tumor microenvironment characteristics. We then quantified each LUAD patient’s TME signature using single-sample gene set enrichment analysis (ssGSEA). Subsequently, we obtained the TME properties of each TCGA-LUAD patient, classified as immune-enriched, fibrotic (IE/F), immune-enriched, nonfibrotic (IE), fibrotic (F), or immune-depleted (D) [26]. PD-L1 protein expression was detected through reverse-phase protein array (RPPA) analysis, and the data were obtained from The Cancer Proteome Atlas (TCPA, http://tcpaportal.org). Furthermore, we assessed the correlation between SPOCK1 and immune cell infiltration using the TIMER database (http://timer.cistrome.org/).

### Analysis of tissue immunofluorescence

Formalin-fixed and paraffin-embedded tissue specimens were selected. 5 µm sections were taken after paraffin-embedding the specimens, and after dewaxing, rehydration and antigen recovery, the primary antibodies added before BSA blocking were treated with EDTA antigen repair buffer (pH 8.0). Sections were incubated overnight in a wet chamber at 4 °C, washed 3 times, and the appropriate type of HRP-labeled secondary antibody was dropped into the overlying tissue and incubated at room temperature for 50 min. After DAPI re-staining of cell nuclei, the films were sealed and photographed under a microscope. All immunofluorescence (IF) sections were analyzed with ImageJ software.

### Exploration and validation of small molecule drug candidates for SPOCK1

To compare the IC_50_ values of different drugs in high- and low-SPOCK1 groups, including common chemotherapeutic drugs, we utilized the “oncoPredict” R package. The “oncoPredict” R package was used to determine the half-maximal IC_50_, which represents the concentration at which a drug achieves 50% inhibition in cell lines. The Wilcoxon signed-rank test was employed for this analysis. Additionally, we utilized the connectivity map (CMap) database (https://clue.io/), which contains the world's largest perturbation-driven gene expression dataset. Using the CMap database, we screened for potential small molecule drugs targeting SPOCK1.

### Xenograft mouse model

LUAD cell lines were injected subcutaneously into 8-week-old male C57BL/6 mice (n = 6 per group). On day 7 after tumor cell injection, VER-155008 (10 mg/kg) or PBS was administered every 2 days starting on day 10 after tumor cell injection and tumor volume was measured every 4 days. Mice were executed 30 days after implantation, and tumor weights were recorded.

### Cell counting /MTS assay

We inoculated lung adenocarcinoma cells in 96-well plates according to 3 × 10^3^ per well overnight. After that, we added 2 μM paclitaxel or 10 μM VER-155008 respectively and incubated for 24 h, 48 h and 72 h. After that, MTS reagent (Promega) was added and incubated for 4 h and OD values were determined.

### Pan-cancer analysis of SPOCK1

We downloaded the uniformly standardized pan-cancer dataset and the associated clinical information from the UCSC (https://xenabrowser.net/) database. Incomplete survival information and survival status samples were removed from the pan-cancer expression data and clinical information to obtain collated high-quality prognostic expression data. The prognostic value of SPOCK1 in different cancer types was assessed by four clinical outcomes: overall survival (OS), disease-specific survival (DSS), disease-free interval (DFI) and progression-free interval (PFI). When p < 0.05 was considered statistically significant.

### Statistical analysis

In this study, the Wilcoxon rank sum test or Student's two-sided t-tests were used to calculate the significance of the differences between the two groups of patients. Kaplan−Meier survival analysis and the log-rank test were performed to assess the statistical significance between the high-SPOCK1 and low-SPOCK1 groups using R language packages (survival, survminer, and ggplot2). Independent prognostic factors were determined by univariate and multivariate Cox regression analysis and were provided by the “forestplot” package in R. Chi-square tests were used to analyze categorical variables. All statistical analyses were performed using R software (version 4.1.2) or Prism 9.0 (GraphPad, San Diego, CA, USA). Each experiment was repeated three or more times and all data were expressed as mean ± standard deviation (SD). Statistical significance is described as follows: ns, not significant; * p < 0.05; * * p ≤ 0.01; * * * p ≤ 0.001.

## Results

### WGCNA analysis identifies key modules associated with TIDE and EMT in lung adenocarcinoma

**Fig. 1:**
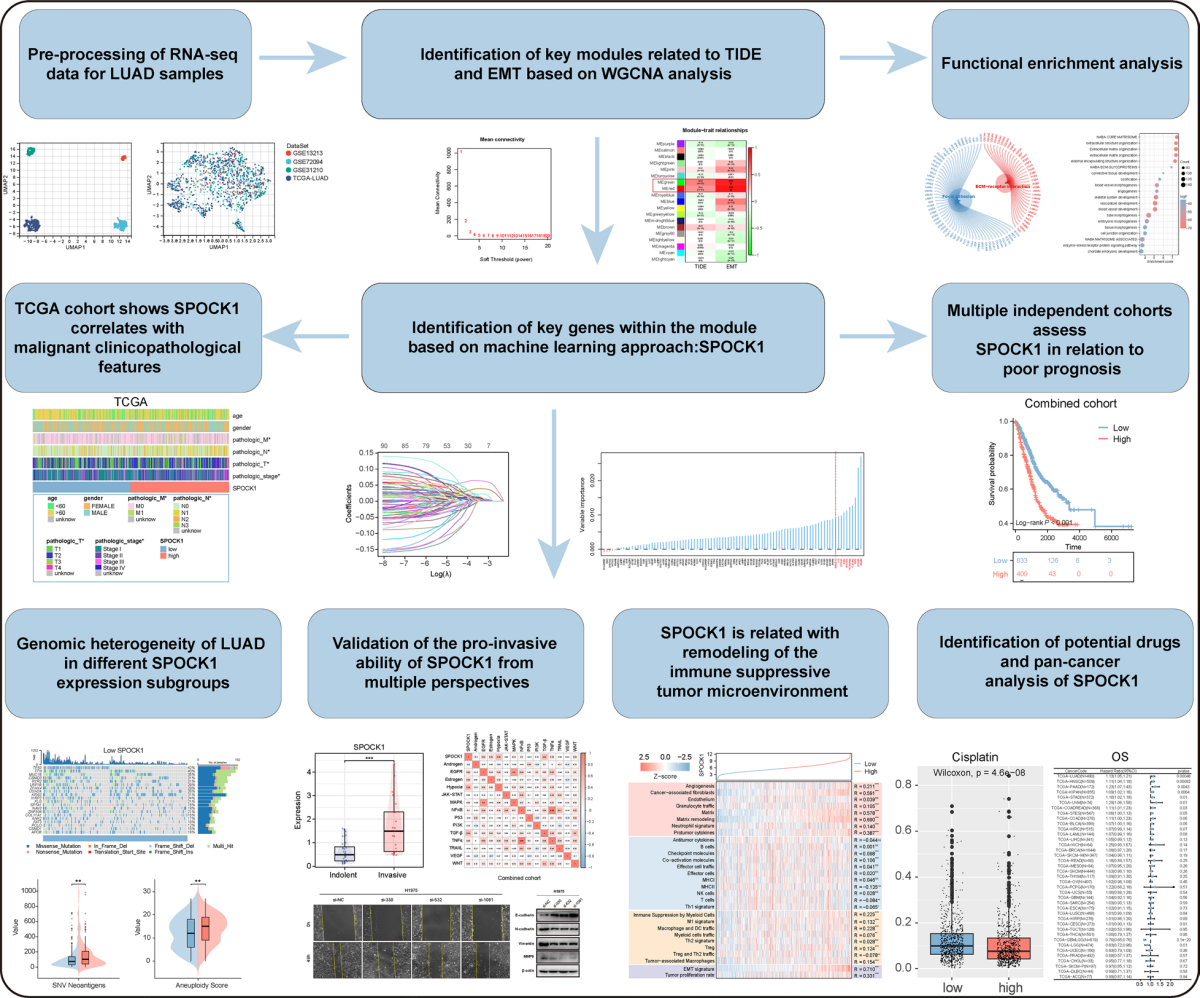
Overview of the study design for the identification of SPOCK1, a key gene associated with the occurrence of EMT and immune evasion phenotype in the tumor microenvironment in lung adenocarcinoma

We first summarize the workflow of our study design, which includes the screening process of SPOCK1 and the exploration of its biological role (Fig. 1). The mRNA expression profiles from LUAD samples in TCGA and GEO databases were merged and globally batch-corrected (Additional file 1: Fig. S1A, B).

**Fig. 2:**
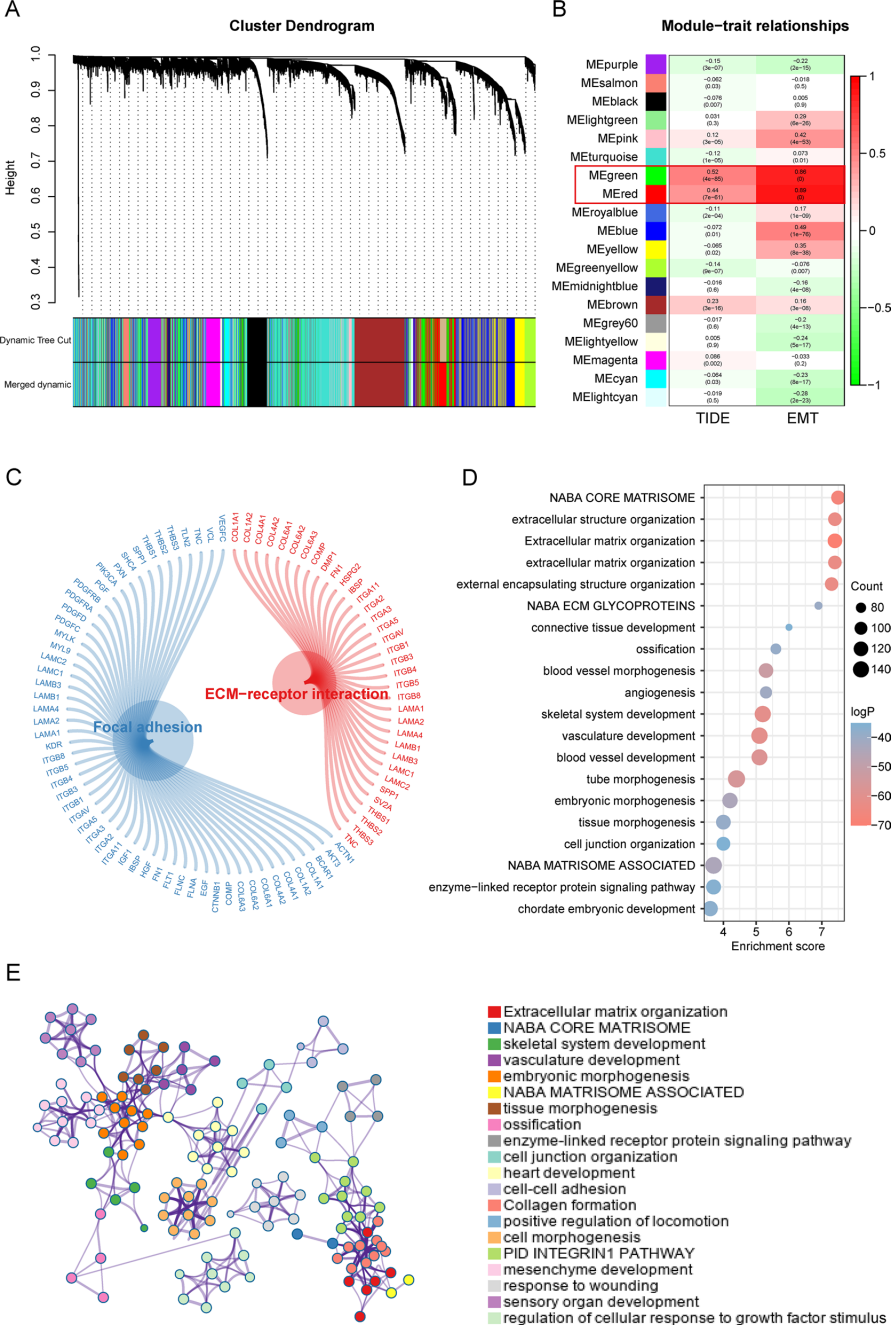
Construction of key gene modules and annotation of biological functions and pathways. **A** A clustering dendrogram formed by weighted correlation coefficients, clustering genes with similar expression patterns into co-expression modules, with each color representing a module. **B** Heatmap of the correlation between module eigengenes (MEs) and TIDE score as well as EMT score. **C**–**E** The top biological pathways of the red and green module genes

Based on the differential gene expression in the combined cohort, we ranked the genes and selected the top 75% for further analysis. To construct a scale-free network, we chose a soft thresholding power of β = 3 (Additional file 1: Fig. S2A–C). The genes were then divided into 19 modules using average linkage clustering based on their expression patterns (Fig. 2A). Further analysis revealed that the green and red modules were highly correlated with both TIDE score and EMT score. Specifically, the green module showed high correlation with TIDE score (R = 0.52, p = 4e-85) and EMT score (R = 0.86, p = 0), while the red module displayed high correlation with TIDE score (R = 0.44, p = 7e-61) and EMT score (R = 0.89, p = 0) (Fig. 2B). The KEGG pathway analysis demonstrated that these genes were associated with extracellular matrix receptor (ECM-receptor) interactions and focal adhesion. The biological functional analysis showed that these genes mainly participate in biological pathways such as extracellular structural organization, extracellular matrix organization, and angiogenesis (Fig. 2C–E).

### Machine learning identifies SPOCK1 as a key gene in lung adenocarcinoma

**Fig. 3:**
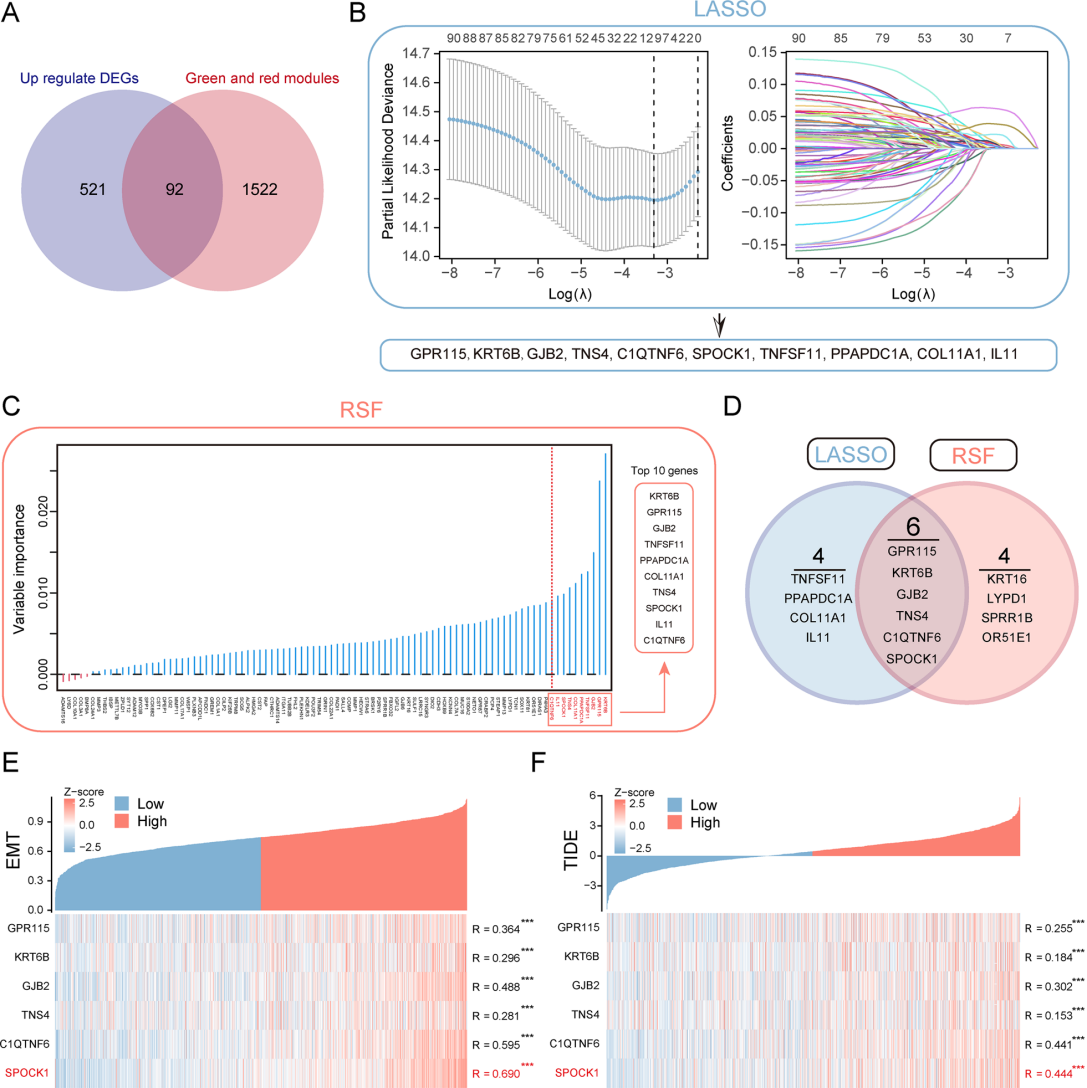
Identification of SPOCK1 as a prognostic key gene within the key modules. **A** Venn plot of the intersection of differentially expressed genes (DEGs) that are abnormally upregulated in tumors and genes within the red and green modules. **B** Determination of the optimal lambda when the partial likelihood of deviance reaches a minimum. And LASSO coefficient of module candidate gene profiles. **C** Screening of the top 10 prognosis-related genes in terms of relative importance using the stochastic survival forest algorithm. **D** Intersection Venn plot of prognostic key genes. Hub genes’ correlation heatmap with (**E**) EMT score and (**F**) TIDE score. (***p < 0.001; **p < 0.01; *p < 0.05; *ns.* no significance)

We identified 613 differentially expressed genes (DEGs) that were upregulated in tumor tissues compared to normal samples (Additional file 2: Table S1). We overlapped these significantly upregulated DEGs with genes within the red and green modules, resulting in the identification of 92 genes (Fig. 3A). Further analysis using LASSO-Cox analysis (Fig. 3B) and Random Survival Forest (RSF) analysis (Fig. 3C) led to the identification of 10 prognosis-related key genes highly associated with patient overall survival (OS). By further overlapping these genes, we obtained 6 genes (Fig. 3D). Subsequent correlation analysis revealed a strong correlation between the SPOCK1 gene and both TIDE score and EMT score (Fig. 3E, F). Therefore, we further analyzed the potential biological roles of the SPOCK1 gene in LUAD patients.

### SPOCK1 is associated with malignant clinicopathological features

**Fig. 4:**
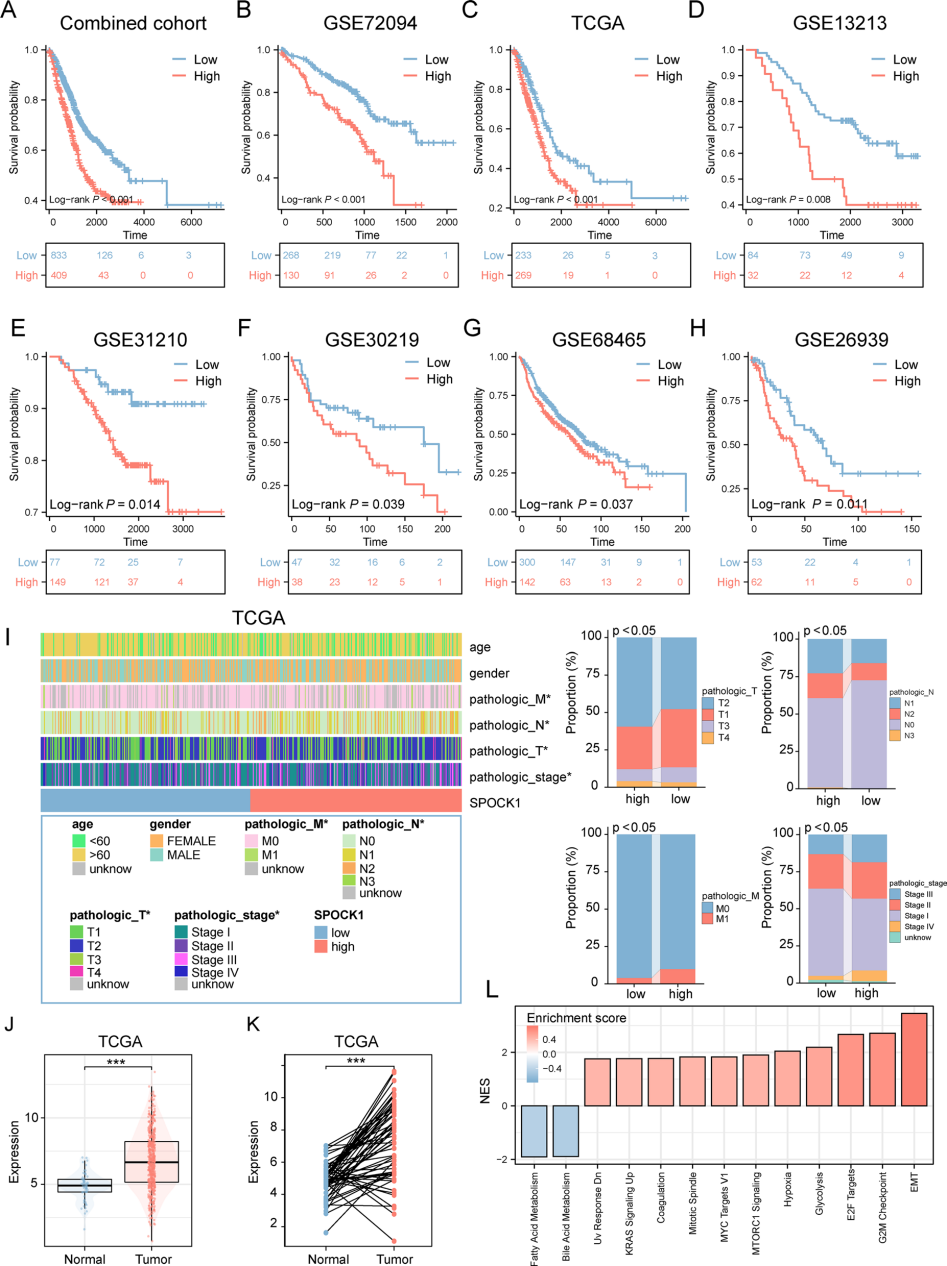
SPOCK1 is associated with adverse clinical outcomes. **A**–**H** Prognostic value of SPOCK1 was assessed in the combined cohort and seven independent cohorts. **I** Heat map of the distribution of SPOCK1 with clinicopathological features. **J** Differential expression of SPOCK1 in normal and tumor tissues. **K** Differential expression of SPOCK1 between normal tissues and paired tumor tissues. **L** Differential expression of SPOCK1 in normal and tumor tissues. **L** GSEA analysis showing the activation profile of biological pathways in the high SPOCK1 group. (***p < 0.001; **p < 0.01; *p < 0.05; ns. no significance.)

We further validated the prognostic value of SPOCK1 in the combined cohort and seven independent LUAD cohorts. As shown in (Fig. 4A–H), SPOCK1 showed a robust prognostic predictive ability in different independent data sets. Combined analysis with clinicopathological features also showed that patients with high SPOCK1 expression tended to exhibit more advanced pathological staging (Fig. 4I). Univariate Cox analysis showed that SPOCK1 was statistically significantly associated with OS in patients with LUAD (HR = 1.141, p < 0.001), while SPOCK1 remained an independent predictor of OS in patients with LUAD (HR = 1.125, p < 0.001) after adjusting for other confounding variables (Additional file 1: Fig. S3A). After that, we selected independent prognostic indicators of OS from multivariate Cox regression and integrated them to construct a prediction model. This model showed excellent agreement between predictions and observations of the 1 year, 3 year, and 5 year probabilities of OS occurrence (Additional file 1: Fig. S3B, C). This implies that the prediction model constructed based on SPOCK1 is very accurate. Consistently, SPOCK1 was significantly more highly expressed in the tumor region compared to the normal group (Fig. 4J, K). Similar results were validated in cell lines, where lung adenocarcinoma cell lines showed higher expression of SPOCK1 than the normal lung epithelial cell line BEAS-2B (Additional file 1: Fig. S3D). Further, in terms of biological pathways, SPOCK1 was mainly involved in EMT pathway, G2M checkpoint and other malignant biological pathways related to cell growth (Fig. 4L). In conclusion, these results suggest that SPOCK1 is highly associated with tumor malignant progression.

### Genomic-level variation of SPOCK1 in lung adenocarcinoma

**Fig. 5:**
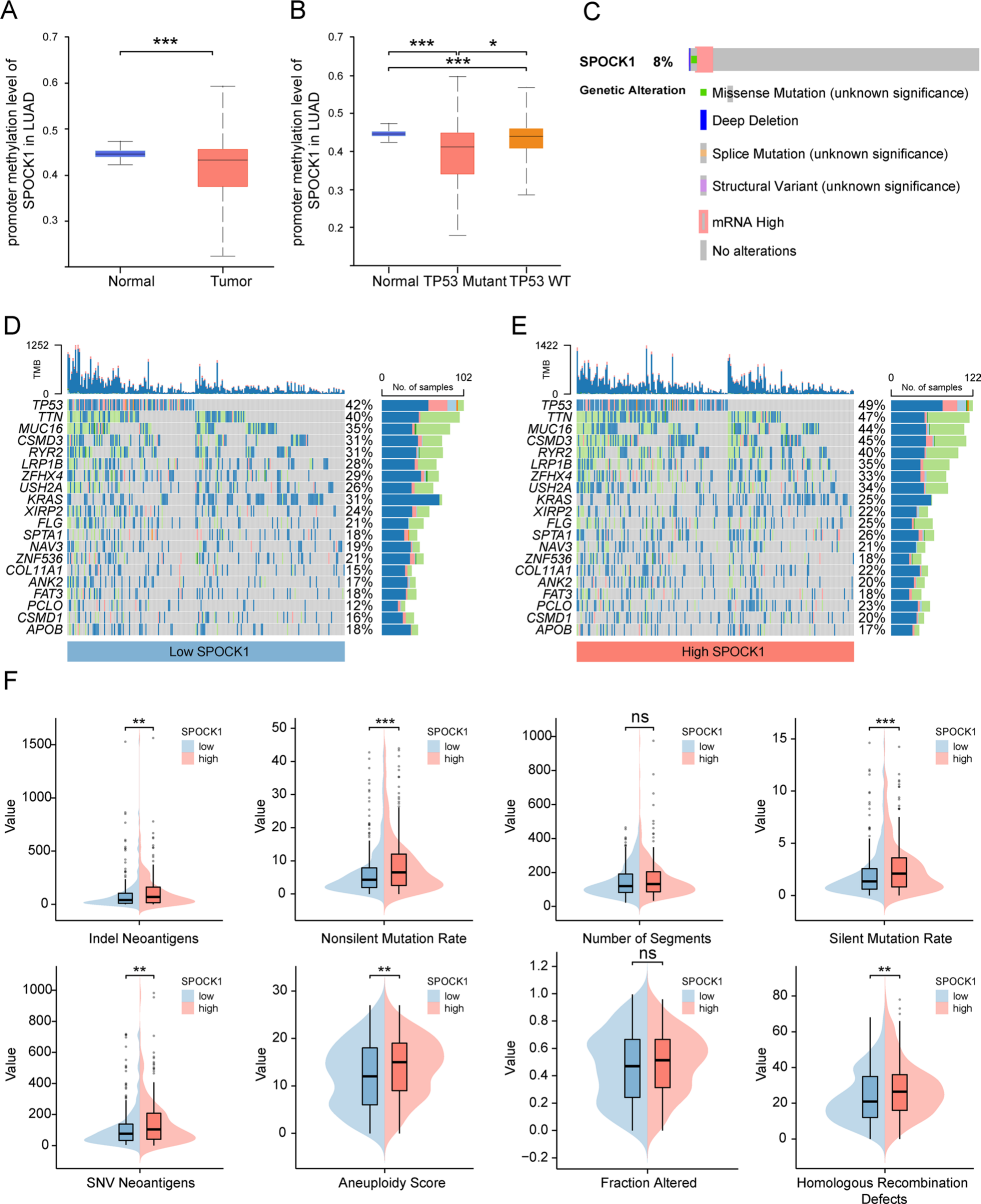
Relationship of SPOCK1 with methylation and mutation. Boxplot indicates the extent of variation in promoter methylation levels in LUAD samples from the UALCAN database, (**A**) in normal and tumor, (**B**) TP53 mutant and TP53 wild-type groups. **C** Exploration of the mutational landscape of SPOCK1 in LUAD using the cBioPortal database. Mutation waterfall plots of the top 20 genes in the (**D**) high SPCOK1 group and (**E**) low SPCOK1 group in terms of mutation frequency. **F** Boxplots show the difference of DNA damage measures between different levels of SPOCK1 expression groups (Wilcon rank-sum test). (***p < 0.001; **p < 0.01; *p < 0.05; ns. no significance.)

We analyzed the changes in the methylation levels of the SPOCK1 promoter in LUAD patients using the UALCAN database. The results demonstrated a significant decrease in the promoter methylation levels of SPOCK1 in tumor patients compared to the normal group. This is consistent with the higher mRNA expression levels of SPOCK1 in tumor patients compared to the normal group (Fig. 5A). Similarly, we also observed a significant decrease in the methylation levels of SPOCK1 in the TP53-mutant group (Fig. 5B), suggesting a link between TP53 mutation and SPOCK1 methylation variation. Analysis from the cBioPortal database showed an 8% mutation rate of SPOCK1 in the LUAD population, Mutation types that promote elevated mRNA expression are most frequently (Fig. 5C). Moreover, we further analyzed the mutation landscape of the top 20 genes with the highest mutation frequencies in the high and low SPOCK1 expression groups. The results showed a significant enrichment of mutations in these highly mutated genes in patients with high SPOCK1 expression (Fig. 5D, E). Consistently, we found that the high SPOCK1 expression group also displayed higher scores in aneuploidy, indel neoantigens, nonsilent mutation rate, silent mutation rate, homologous recombination defects, SNV neoantigens, and fraction altered (Fig. 5F). Overall, these findings partially explain the adverse survival outcomes observed in patients with high SPOCK1 expression.

### Malignant biological characteristics of SPOCK1 in lung adenocarcinoma

**Fig. 6:**
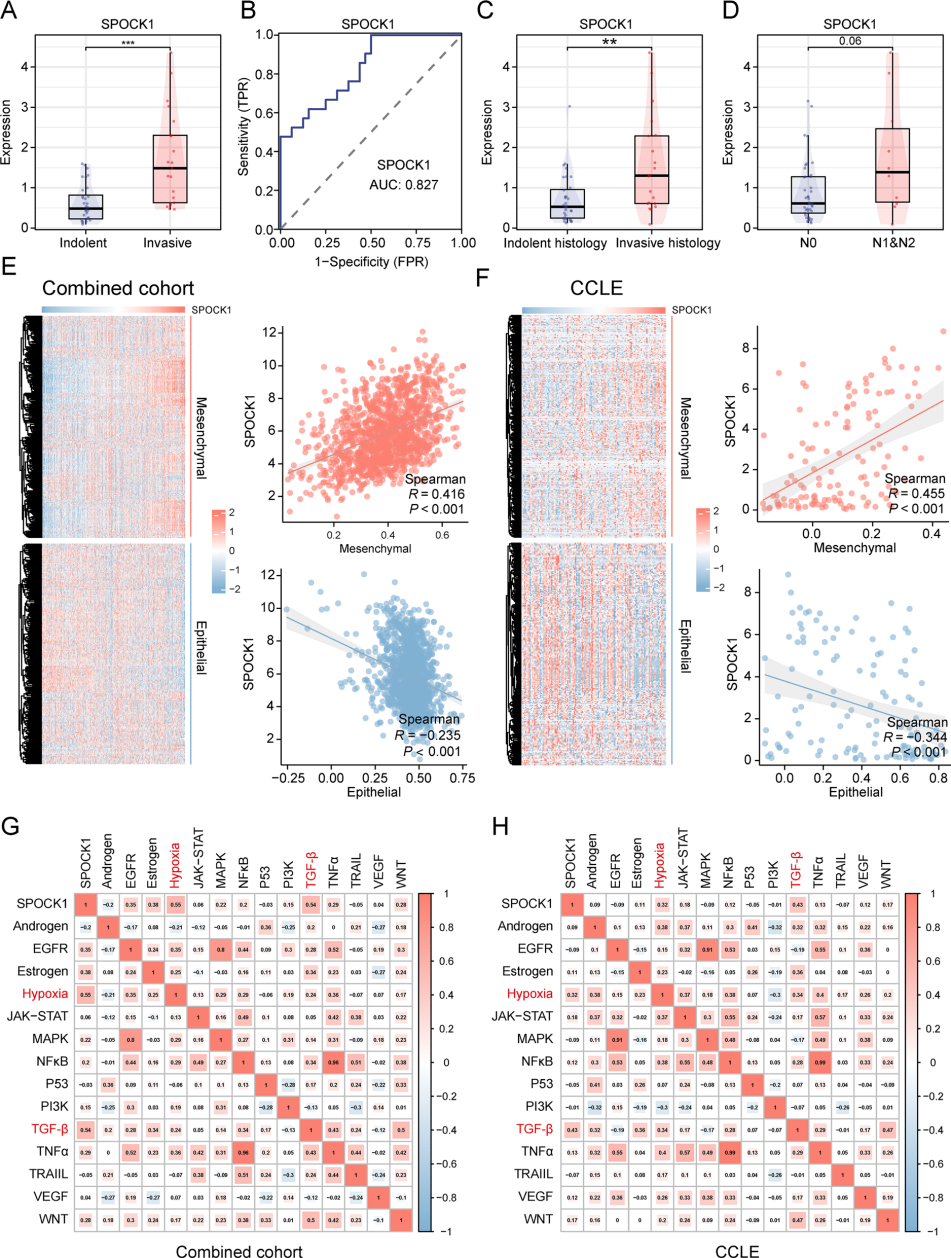
SPOCK1 is associated with invasive properties. In the GSE166722 dataset, (**A**) SPOCK1 expression levels in invasive phenotype and inert phenotype LUAD. **B** ROC curves showing the diagnostic efficacy of SPOCK1 in differentiating between aggressive and inert patients. **C** Differential expression levels of SPOCK1 in different aggressive pathological histological subtypes; (**D**) Differential expression levels of SPOCK1 in patients with or without lymph node metastasis. **E** Heatmap of the distribution of SPOCK1 and EMT-related markers in the combined cohort and (**F**) NSCLC cell lines, and the mesenchymal score and epithelial score were assessed based on the ssSGEA algorithm, and correlations were calculated (spearman test). **G** The potential association between SPOCK1 and dysregulated oncogenic pathways in the combined cohort and (**H**) NSCLC cell lines. (***p < 0.001; **p < 0.01; *p < 0.05; *ns.* no significance.)

To further understand the association between SPOCK1 and malignant features of LUAD, we initially analyzed and compared the expression differences of SPOCK1 between the indolent and invasive subtypes in the GSE166722 dataset. The results indicated a significant upregulation of SPOCK1 expression in the invasive subtype compared to the indolent subtype (Fig. 6A). Similarly, the ROC curve demonstrated the efficiency of SPOCK1 in distinguishing between the indolent and invasive subtypes (AUC = 0.827) (Fig. 6B). Furthermore, SPOCK1 also exhibited higher expression levels in invasive histological subtypes and patients with lymph node metastasis (Fig. 6C, D). Considering the link between SPOCK1 and EMT-related pathways, we analyzed the correlation of SPOCK1 with mesenchymal and epithelial markers in the combined cohort and NSCLC cell lines from CCLE. The results showed a positive correlation between SPOCK1 expression and mesenchymal markers, while a negative correlation was observed with epithelial markers (Fig. 6E, F). Regarding carcinogenesis pathways, we found a significant association between SPOCK1 expression and activation of the TGF-β pathway and upregulation of the Hypoxia pathway, both at the histological and cellular levels (Fig. 6G, H).

### *SPOCK1 promotes *in vitro* migration and invasion of lung adenocarcinoma cells *via* EMT*

**Fig. 7:**
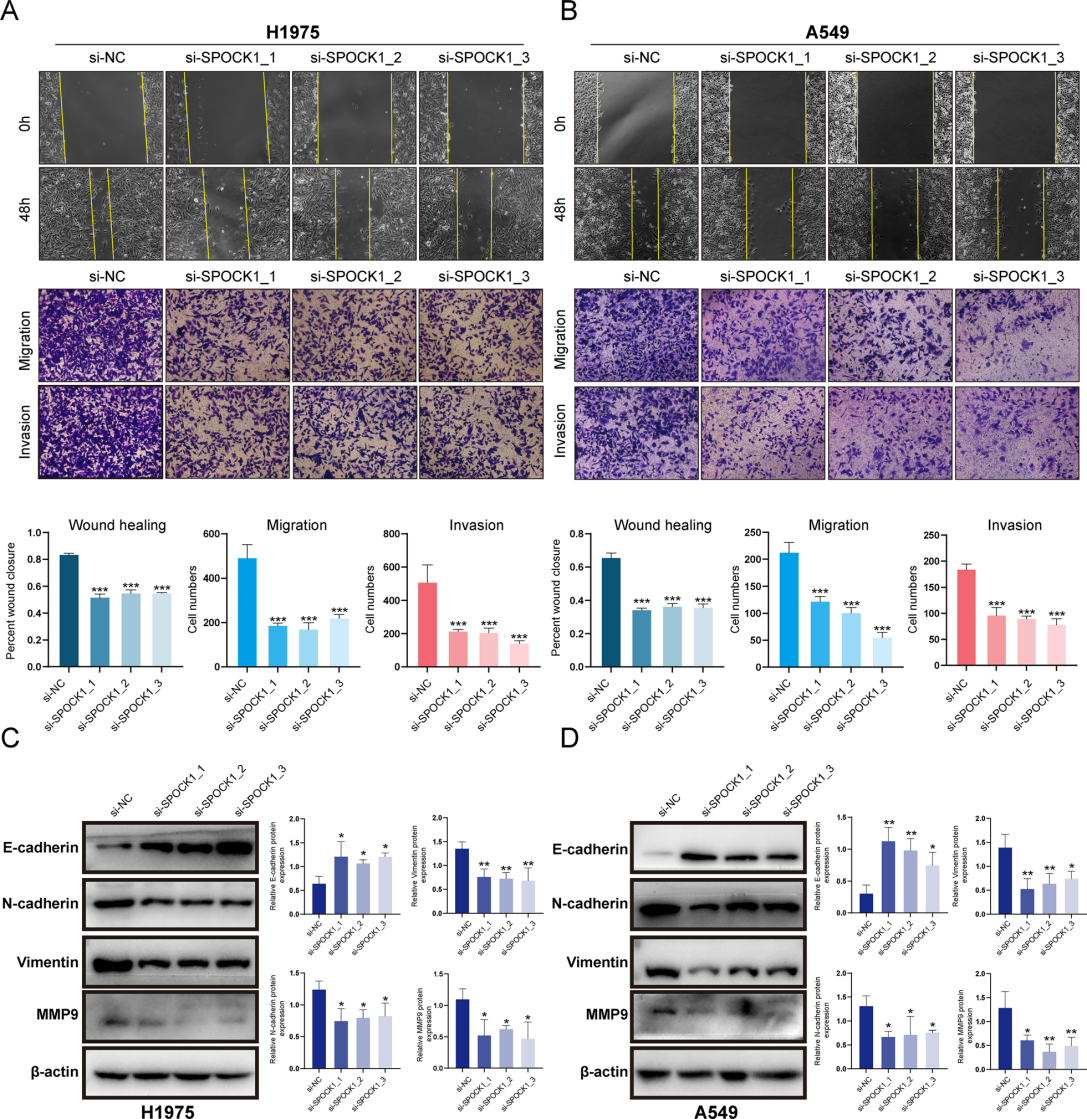
SPOCK1 promotes in vitro migration and invasion of lung adenocarcinoma cells. (**A**–**B**) Images of wound healing and transwell assay assays in the NC group and SPOCK1 knockdown group. (**C**–**D**) Expression of EMT-related marker and MMP9 was verified by Western blotting assay assay in LUAD cells transfected with siRNA. (***p < 0.001; **p < 0.01; *p < 0.05)

In vitro experiments were conducted to knockdown SPOCK1 in lung adenocarcinoma cell lines A549 and H1975 (Additional file 1: Fig. S4A, B). Both the wound healing assay and the transwell assay (including migration and invasion assays) demonstrated that knockdown of SPOCK1 significantly inhibited the migration and invasion abilities of lung adenocarcinoma cells (Fig. 7A, B). As for the regulation of EMT, the expression of epithelial marker, E-cadherin, in A549 and H1975 was upregulated after the knockdown of SPOCK1, while the expression of the mesenchymal markers, that is, N-cadherin, vimentin was downregulated; furthermore, it is well known that matrix metalloproteinases (MMPs) have an important role in tumor progression and metastasis [27], our results also indicated that knockdown of SPOCK1 significantly decreased the expression of MMP9 (Fig. 7C, D). Overall, these findings highlight the key role of SPOCK1 in promoting the malignant invasive characteristics of lung adenocarcinoma cells.

### SPOCK1 is associated with the formation of immunosuppressive tumor microenvironment in lung adenocarcinoma

**Fig. 8:**
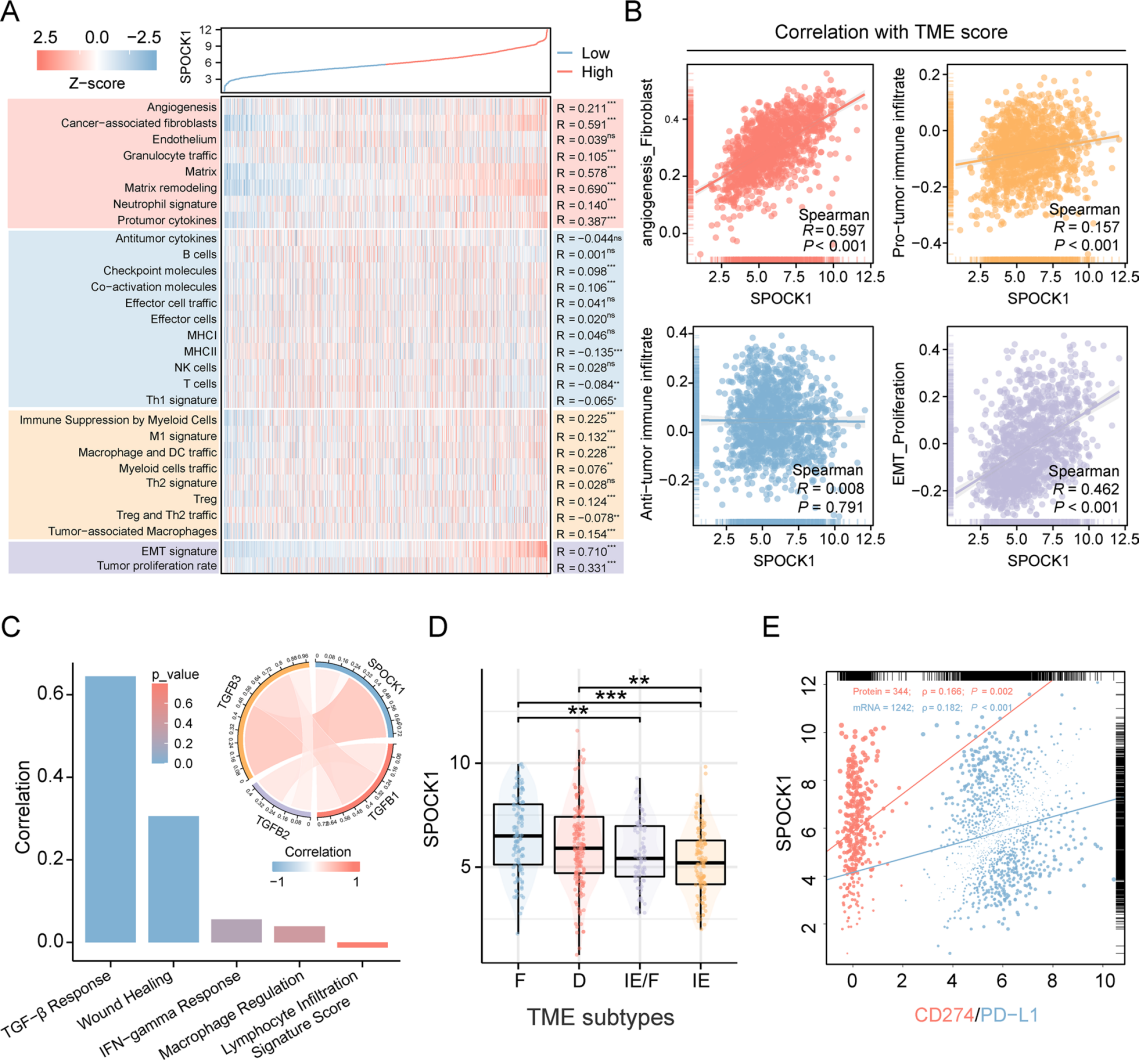
Role of SPOCK1 in the tumor microenvironment. **A** Heatmap of the distribution of 29 TME associated signatures with SPOCK1 expression values. **B** Scatter plot of the correlation between 4 TME scores and SPOCK1 expression values. **C** Histogram of correlation between SPOCK1 and 5 immune features and chord plot of correlation with TGF-β1 (TGFB1), TGF-β2 (TGFB2), and TGF-β3 (TGFB3) (spearman test). **D** Distribution of SPOCK1 expression in TME subtypes defined by Bagaev et al. **E** Correlation between SPOCK1 and CD274 (mRNA level) and PD- L1 (protein level, from TCPA database) correlation scatter plots. (***p < 0.001; **p < 0.01; *p < 0.05; ns. no significance.)

To understand the potential association between SPOCK1 and tumor microenvironment (TME) remodeling, we evaluated the distribution differences of 29 TME signatures in the high and low SPOCK1 expression groups using the ssGSEA algorithm. The results showed that SPOCK1 was primarily associated with angiogenesis, matrix remodeling, and matrix-related pathways. In terms of immune infiltration, SPOCK1 exhibited a positive correlation with the infiltration of cancer-associated fibroblasts (CAF), myeloid-derived suppressor cells (MDSCs), tumor-associated macrophages (TAMs), and regulatory T cells (Tregs) (Fig. 8A). Further categorizing these 29 signatures into four TME scores, we found that SPOCK1 was highly correlated with the angiogenesis_fibroblast, pro-tumorigenic immune infiltration, and EMT_Proliferation TME scores, whereas no correlation was observed with the anti-tumorigenic immune infiltration TME score (Fig. 8B).

Next, we analyzed the correlation between SPOCK1 and 5 known immune signature scores. The results showed a strong correlation between SPOCK1 and the TGF-β response score. The chord diagram demonstrated the correlation between SPOCK1 and TGF-β1, TGF-β2, and TGF-β3 in the TGF-β pathway (Fig. 8C). In terms of the TME subtypes defined by Bagaev et al., SPOCK1 exhibited active expression in the F subtype and the D subtype, while downregulated expression was observed in the IE subtype. These findings suggested a potential link between SPOCK1 and the formation of immunosuppressive TME (Fig. 8D). Furthermore, considering the association between SPOCK1 and the immunosuppressive microenvironment, we analyzed the correlation between SPOCK1 and the mRNA and protein levels of PD-L1. The results showed a positive correlation between SPOCK1 and PD-L1 expression at both the mRNA and protein levels (Fig. 8E). This suggests a potential regulatory role of SPOCK1 in shaping the immunosuppressive microenvironment through PD-L1.

### ***SPOCK1 negatively correlates with CD8***^+^***T-cell infiltration in lung adenocarcinoma***

**Fig. 9:**
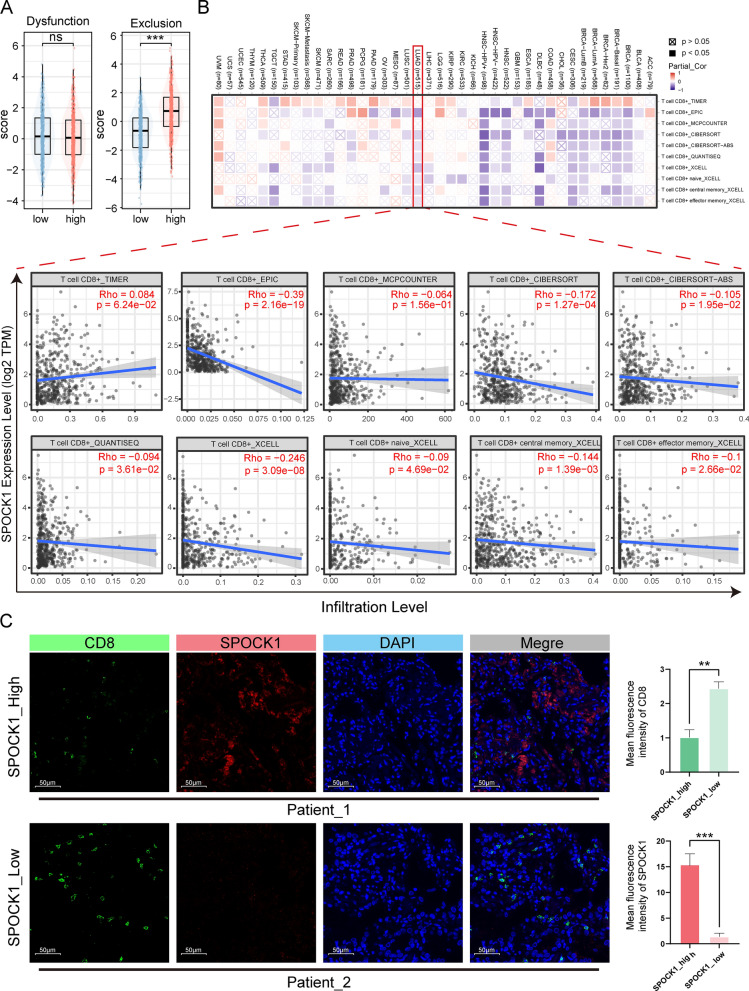
Highly expressed SPOCK1 is associated with low infiltrating CD8^+^ T cells. **A** Differences in the distribution of T cell rejection and dysfunction scores in high and low SPOCK1 groups. **B** The relationship between SPOCK1 and CD8^+^ T cell infiltration was assessed by the TIMER database using multiple immune infiltration methods. **C** Representative tissue immunofluorescence images for SPOCK1 and CD8. (SPOCK1_High, n = 3; SPOCK1_Low, n = 3). Scale bar = 50 μm. (***p < 0.001; **p < 0.01; *p < 0.05; *ns.* no significance.)

The TIDE score, consisting of the T cell dysfunction score and T cell exclusion score, is used to assess T cell function in the tumor microenvironment. We analyzed the relationship between SPOCK1 and the T cell dysfunction score and T cell exclusion score. Our results showed that patients in the high SPOCK1 group often had higher T cell exclusion scores (Fig. 9A). Then, using the TIMER database, we further investigated the correlation between SPOCK1 in pan-cancer and the infiltration levels of CD8^+^ T cells using multiple immune infiltration evaluation methods. The analysis showed that SPOCK1 was negatively correlated with CD8^+^ T cell infiltration in most cancers (Fig. 9B). Next, we selected samples from clinical patients and used immunofluorescence staining to examine the distribution relationship between SPOCK1 and CD8^+^. The results also showed that tissue samples with high SPOCK1 expression had relatively fewer infiltrating CD8^+^ T cells (Fig. 9C, Additional file 1: Fig. S5).

### Identification and validation of the potential drugs for SPOCK1 in lung adenocarcinoma

**Fig. 10:**
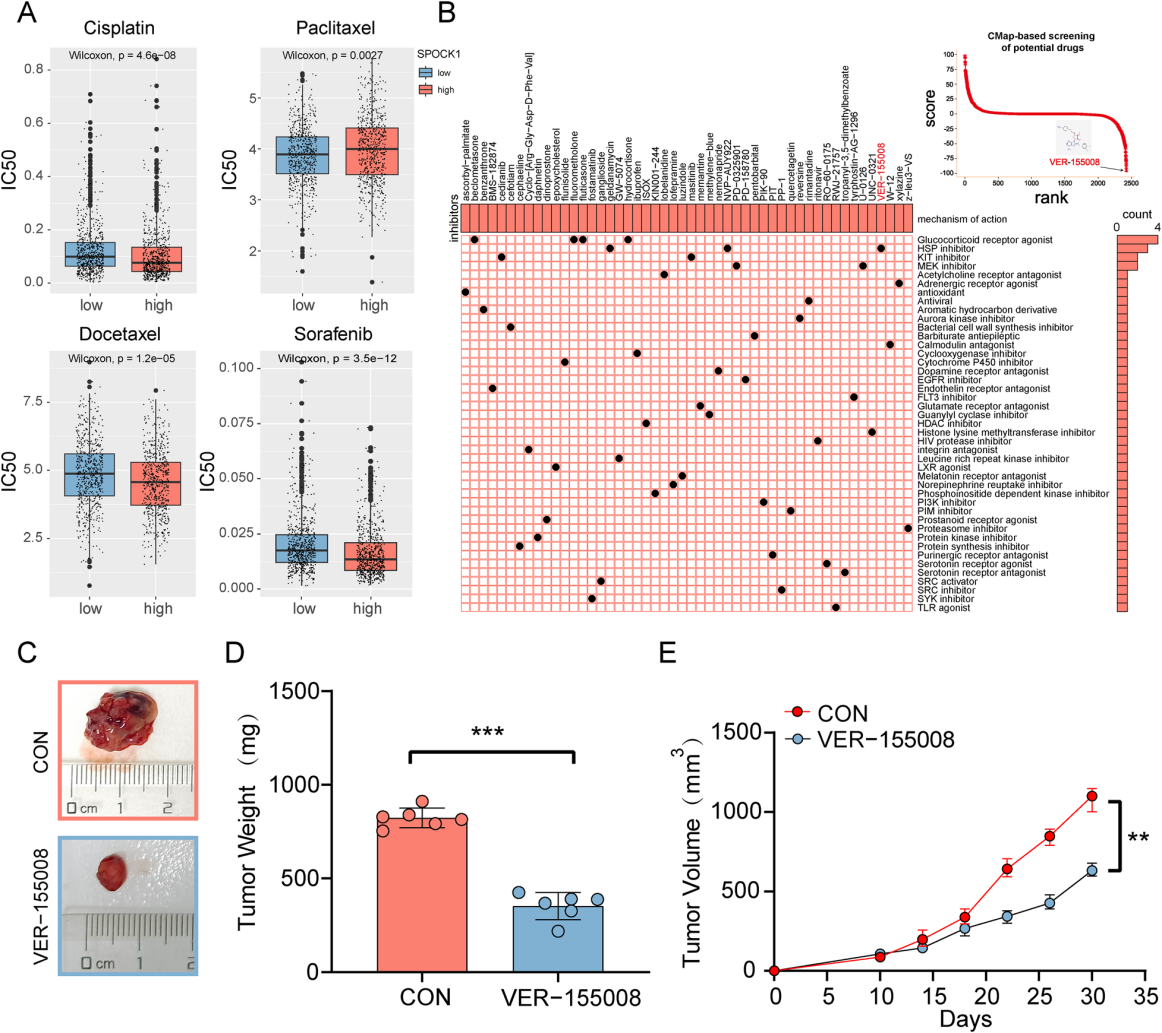
Identification of potential drugs for the treatment of SPOCK1. **A** Therapeutic drugs showed significant IC50 differences in high- and low-SPOCK1 groups. **B** CMap-based screening of the potential drugs for SPOCK1. **C** Subcutaneous transplantation tumor isolates. (D) Tumor weight of mice after subcutaneous injection with PBS (n = 6) or VER-155008 (n = 6). **E** Tumor growth of subcutaneous LUAD tumors over time. Data are expressed as mean ± SD (***p < 0.001; **p < 0.01; *p < 0.05; *ns.* no significance.)

In order to explore the potential clinical value of SPOCK1, we conducted further analysis on its relationship with several commonly used chemotherapy drugs for lung cancer. The results showed that patients with high SPOCK1 expression had increased sensitivity to cisplatin, docetaxel, and sorafenib, while those with low SPOCK1 expression were more responsive to paclitaxel (Fig. 10A). Combination therapy is a widely used approach in clinical cancer treatment. Taking into consideration the pro-tumor characteristics of SPOCK1, we utilized CMap to screen potential drugs based on the differential expression profile between high and low SPOCK1. We visualized the top 50 candidate small molecules, among which VER-155008 had the most significant disruptive effect on SPOCK1 expression profile (Fig. 10B). Considering, the SPOCK1 expression of H1975 was higher than that of H1299 (Additional file 1: Fig. S3D). Thus, we defined H1975 as SPOCK1_High (S_H) cells, and we defined H1299 as SPOCK1_Low (S_L), and evaluated the role of SPOCK1 expression in drug sensitivity in vitro. Cell proliferation assays showed that after administration of VER-155008 and Paclitaxel, cell proliferation began to be down-regulated over time. Consistent with previous analyses, at 72 h, VER-155008 is going to be significantly more effective at inhibiting H1975(S_H) than H1299(S_L). On the contrary, the inhibitory effect of Paclitaxel on H1975(S_H) was significantly weaker than that of H1299(S_L) cells (Additional file 1: Fig. S6A, B). Afterwards, considering the heterogeneity that exists between tumor cells. We further analyzed the differences in the killing effect of VER-155008 on cells with or without spock1 knockdown on the H1975 cell line. We found that at 72 h, the killing effect of VER-155008 was attenuated for the SPOCK1 knockdown group compared to the control group (Additional file 1: Fig. S6C, D). This result somewhat suggests the role of SPOCK1 as a candidate indicator of drug sensitivity. Subsequently, a xenograft mouse model was established and treated with the VER-155008 small molecule compound. The results demonstrated that VER-155008 significantly inhibited tumor growth (Fig. 10C). Compared to the control group, the VER-155008-treated group exhibited a significant reduction in both the volume and weight of the subcutaneous tumors (Fig. 10D, E), Interestingly, we found that SPOCK1 expression was also decreased in the VER-155008-treated group. This suggests a potential mechanism of cancer suppression by VER-155008 (Additional file 1: Fig. S6E).

### The overview of SPOCK1 in human cancers

Due to the limited research on SPOCK1 in cancer, we evaluated its expression at a pan-cancer level. Overall, SPOCK1 exhibited differential expression across various cancer types. It showed downregulation in cancers such as KICH, KIRC, and KIRP. Conversely, abnormal upregulation was observed in cancers including LIHC, LUAD, LUSC, and PRAD (Additional file 1: Fig. S7A). These findings suggest the involvement of SPOCK1 in tumorigenesis processes. Subsequent univariate Cox regression analysis demonstrated significant prognostic value of SPOCK1 in cancers such as LUAD, KIRC, and PAAD (Additional file 1: Fig. S7B).

## Discussion

Recent studies have shown a close relationship between EMT and immune escape. It has been reported that the formation of the inflammatory microenvironment in lung adenocarcinoma is highly related to the status of tumor EMT [28]. Alsulman et al. confirmed the involvement of EMT in PD-L1-mediated immune escape in breast cancer [29]. In addition, Wang et al. and their colleagues characterized the crosstalk between EMT and immune escape based on comprehensive multi-omics studies [30]. These studies have all revealed the coexistence between EMT and immune escape. In this study, we explored the key modules related to immune escape and EMT through WGCNA analysis, followed by the selection of hub genes and survival analysis. Finally, SPOCK1 was identified as a therapeutic target related to LUAD prognosis and the immune suppressive microenvironment.

We conducted a comprehensive evaluation of SPOCK1 in multiple independent cohorts of LUAD. The high SPOCK1 expression group showed significantly shorter overall survival compared to the low SPOCK1 expression group. This finding is consistent with the previous study by Váncza et al. on the prognostic value of SPOCK1 in ovarian cancer [19]. The combination of clinical and pathological features, as well as the univariate and multivariate Cox analysis, also demonstrated SPOCK1 as an independent risk factor. Furthermore, the heatmap including SPOCK1 expression and tumor stage exhibited good predictive performance for prognosis. Moreover, the high SPOCK1 expression group showed enrichment of aggressive clinical pathological features and a higher mutation frequency, which partly explains the adverse prognosis associated with SPOCK1. The pan-cancer analysis from the TCGA cohort also showed a correlation between SPOCK1 overexpression and poor prognosis in the majority of tumors.

The regulation of tumor cell invasion and metastasis by SPOCK1 is an important factor contributing to poor prognosis in LUAD patients. SPOCK1 is considered to be one of the key regulatory genes involved in maintaining the dynamic balance of ECM in the tumor microenvironment. It participates in oncogenic activities that promote abnormal activation of EMT [31]. SPOCK1 has been reported to promote cancer metastasis in various types of cancer. For example, studies have shown that SPOCK1 promotes cancer metastasis in glioma cells through the PI3K/AKT and Wnt/β-catenin signaling pathways [32]. Similarly, the knockdown of the SPOCK1 gene has been shown to suppress the malignant tumor characteristics of colon cancer cells [33]. In our study, we found that knockdown of SPOCK1 effectively inhibits invasion and migration of lung adenocarcinoma cells and significantly suppresses the epithelial-mesenchymal transition process in tumor cells. Additionally, we observed that SPOCK1 is mainly enriched in the invasive subtype of an early-stage lung adenocarcinoma cohort containing invasive subtype information. These results demonstrate the malignant biological characteristics of SPOCK1 in lung adenocarcinoma.

Furthermore, matrix metalloproteinases (MMPs) play a crucial role in cancer cell invasion and metastasis. MMP-9 and MMP-2 are the main matrix metalloproteinases [34]. Studies have indicated that SPOCK1 can promote the abnormal activation of MMP-3, MMP-9, and MMP-2, leading to directed migration of tumor cells from the edge of the tumor mass and ultimately inducing ECM remodeling [35–37]. This is consistent with the results we observed in lung adenocarcinoma cells. It is worth mentioning that research has shown MMP-9's ability to regulate tumor immune surveillance by modulating the expression of PD-L1 [38]. Interestingly, our study also revealed a significant correlation between SPOCK1 and PD-L1 expression. Additionally, at both the tissue and cellular levels, SPOCK1 is closely associated with the hypoxia signaling pathway and TGF-β signaling pathway. The hypoxic microenvironment significantly promotes angiogenesis, the formation of connective tissue, and the generation of inflammation, all of which contribute to tumor progression and the development of treatment resistance [39, 40]. Meanwhile, the TGF-β signaling pathway not only promotes cancer cell invasion and dissemination but also creates an immune suppressive environment by shaping the tumor’s structure and inhibiting the anti-tumor activity of immune cells, thereby impeding or weakening immune-based cancer therapies [41]. In summary, SPOCK1 can promote LUAD invasion and metastasis through various mechanisms, and this may also be one of the reasons for tumor evasion of immune surveillance.

The expression pattern of the tumor microenvironment (TME) plays a crucial role in cancer progression [42]. The imbalance of TME is likely to be a partial cause of the poor prognosis of SPOCK1. It has been reported that the expression of SPOCK1 in colorectal cancer is closely associated with immune infiltration and the expression of CD206, a marker for M2 macrophages [43]. In our study, patients with high SPOCK1 expression showed significant enrichment of immune suppressive components in the tumor microenvironment, including tumor-associated macrophages, myeloid-derived suppressor cells (MDSCs), and regulatory T cells (Treg). Tumor-associated macrophages are considered to drive malignant cancer progression by inducing tumor-associated angiogenesis, promoting tumor invasion, migration, and intravasation [44]. Consistently, more than 80% of studies in one meta-analysis demonstrated a correlation between macrophage infiltration density and poor prognosis in cancer patients [45]. In terms of TME subtypes, SPOCK1 expression was significantly enriched in the fibrotic and immune-depleted subtypes. In summary, the increased presence of these immune suppressive components within the tumor microenvironment may partially explain the poor prognosis of the high SPOCK1 expression group. CD8^+^ T cells infiltrating tumors play a crucial role in mediating anti-tumor immune response and are essential in clinical interventions [46]. In our study, various immune infiltration algorithms and immunofluorescence analysis consistently demonstrated that high expression of SPOCK1 was associated with low infiltration of CD8 + T cells. These results consistently suggest that SPOCK1 is associated with the formation of an immunosuppressive microenvironment in LUAD, however, more studies are still needed to validate the above findings.

Chemotherapy and targeted therapy, as the main treatment methods for LUAD, have significantly prolonged the survival time of patients [2, 47]. However, LUAD easily develops resistance to chemotherapy and targeted therapy. The limited time for treatment correction is due to the loss of tumor sensitivity to drugs [48]. Therefore, improving patients' clinical outcomes can be accomplished by implementing personalized treatment plans and predicting the effectiveness of drug therapy. This study shows that LUAD with different SPOCK1 expression groups exhibit different sensitivities to chemotherapy and targeted therapy. In subsequent in vitro experiments we used paclitaxel to initially validate the feasibility of SPOCK1 as a biomarker for drug sensitivity. Similarly, in glioblastoma, aberrant expression of SPOCK1 was shown to induce Temozolomide resistance [49]. Additionally, VER-155008, the compound with the greatest effect on perturbing SPOCK1 expression profiles, was identified through CMap screening. Reports have indicated that VER-155008, as an HSP70 small molecule inhibitor, promotes apoptosis in colon cancer cells [50] and effectively inhibits the growth of lung cancer cell lines [51]. Our study showed that lung adenocarcinoma cells with high expression of SPOCK1 exhibited sensitivity to VER-15500. Consistently, in vivo experiment demonstrates that VER-155008 can effectively inhibit tumor growth in mice. Interestingly, we found a downregulation of SPOCK1 expression in tumors from the VER-155008 treated group. Previous studies have indicated that upregulation of HSP70 induces TGF-β secretion [52]. Considering the existence of mutual crosstalk between the TGF-β pathway and SPOCK1, we hypothesized that VER-155008 may down-regulate the activation of the TGF-β pathway by inhibiting the expression of HSP70, which finally led to a decrease in the expression of SPOCK1. However, the mechanism involved still needs further experimental exploration.

There are still some limitations of this study. The present study was mainly analyzed using a retrospective cohort, and a prospective cohort of patients is still needed to further validate the findings of this study. Our study also revealed a close relationship between SPOCK1 and in vitro cell migration/invasion, as well as immune microenvironment remodeling in lung adenocarcinoma. These findings suggest that SPOCK1 plays a role in the pathogenesis and prognosis of cancer. However, further research is still required to explore the in vivo effects of SPOCK1.

## Conclusion

The overexpression of SPOCK1 can lead to poor survival outcomes by driving EMT and inducing immune escape of tumor cells. It is suggested that SPOCK1 may be a potential therapeutic target for clinical lung adenocarcinoma.

### Supplementary Information

**Additional file 1: Fig S1.** Pre-processing of LUAD number samples (A) Visualization of clustering results of LUAD samples before and after de-batching using UMAP plots (B) Box plots of expression values of LUAD samples before and after de-batching. **Fig S2.** Gene co-expression network construction. （A） Sample dendrogram and trait indicator. (B) Analyze the scale-free fit index of the 1-20 soft threshold power (β). (C) Analyze the average connectivity of 1-20 soft threshold power. **Fig S3.** The prognostic value of the SPOCK1 in the combined cohort. （A） Univariate and multivariate Cox regression analyses of the association between clinical features and OS of patients. (B) Construction of a nomogram for survival prediction based on SPOCK1. (C) The calibration curve for the nomogram model. Three colored lines (purple, red, and black) represent the performance of the nomogram. A closer fit to the diagonal gray line indicates a better estimation. (D) The western blot of SPOCK1 in normal lung cell lines and lung cancer cell lines. **Fig S4.** Knockdown of SPOCK1 in cell lines. Western blotting assay after knockdown of SPOCK1 in （A）H1975 and （B）A549. **Fig S5.** Immunofluorescence assay for SPOCK1 and CD8 in LUAD samples. **Fig S6.** Validation of the candidate drug for SPOCK1. (A) The proliferation of control, Paclitaxel((2μM)) or VER-155008(10μM) treated lung cancer cells was measured by MTS assay at the indicated time points. (B) The difference in the reduction of proliferation for Paclitaxel((2μM)) or VER-155008(10μM) treated lung cancer cells at 72h, Ratio of relative reduction in proliferation =（OD Value（con）- OD Value（VER-155008 or Paclitaxel））/OD Value（con）. (C) The proliferation of H1975 cells treated in the control group, VER-155008 group, si_SPOCK1_1 group, and si_SPOCK1_1 + VER-155008 group was detected by the MTS method at the indicated time points. (D) The difference in proliferation reduction between control+VER-155008 and si_SPOCK1_1+VER-155008 groups, after 72 hours of treatment of the H1975 cell line. (E) Immunofluorescence assay for SPOCK1 in control (n=6). or VER-155008 treated group (n=6). **Fig S7.** Pan-cancer study of SPOCK1.（A）The mRNA expression of SPOCK1 between tumor and normal tissues was assessed from the TCGA database. （B）Univariate Cox regression analyses estimating prognostic value (OS/PFI/DSS/DFI) of SPOCK1 in pan-cancers from the TCGA database. （***p < 0.001; **p < 0.01; *p < 0.05; ns. no significance.）

**Additional file 2.**

## Data Availability

The original data presented in the study are included in the article/Additional files and further inquiries can be directed to the corresponding authors.
