# Supplementary material for: SPOCK1, as a potential prognostic and therapeutic biomarker for lung adenocarcinoma, is associated with epithelial-mesenchymal transition and immune evasion
Source: J Transl Med. 2023 Dec 12;21:909. doi: 10.1186/s12967-023-04616-3 (PMC10717042; doi:10.1186/s12967-023-04616-3)
Supplement: Supplementary file 1 — Additional file 1: Fig S1. Pre-processing of LUAD number samples (A) Visualization of clustering results of LUAD samples before and after de-batching using UMAP plots (B) Box plots of expression values of LUAD samples before and after de-batching. Fig S2. Gene co-expression network construction. （A） Sample dendrogram and trait indicator. (B) Analyze the scale-free fit index of the 1-20 soft threshold power (β). (C) Analyze the average connectivity of 1-20 soft threshold power. Fig S3. The prognostic value of the SPOCK1 in the combined cohort. （A） Univariate and multivariate Cox regression analyses of the association between clinical features and OS of patients. (B) Construction of a nomogram for survival prediction based on SPOCK1. (C) The calibration curve for the nomogram model. Three colored lines (purple, red, and black) represent the performance of the nomogram. A closer fit to the diagonal gray line indicates a better estimation. (D) The western blot of SPOCK1 in normal lung cell lines and lung cancer cell lines. Fig S4. Knockdown of SPOCK1 in cell lines. Western blotting assay after knockdown of SPOCK1 in （A）H1975 and （B）A549. Fig S5. Immunofluorescence assay for SPOCK1 and CD8 in LUAD samples. Fig S6. Validation of the candidate drug for SPOCK1. (A) The proliferation of control, Paclitaxel((2μM)) or VER-155008(10μM) treated lung cancer cells was measured by MTS assay at the indicated time points. (B) The difference in the reduction of proliferation for Paclitaxel((2μM)) or VER-155008(10μM) treated lung cancer cells at 72h, Ratio of relative reduction in proliferation =（OD Value（con）- OD Value（VER-155008 or Paclitaxel））/OD Value（con）. (C) The proliferation of H1975 cells treated in the control group, VER-155008 group, si_SPOCK1_1 group, and si_SPOCK1_1 + VER-155008 group was detected by the MTS method at the indicated time points. (D) The difference in proliferation reduction between control+VER-155008 and si_SPOCK1_1+VER-155008 groups, after 72 hours of trea [file 12967_2023_4616_MOESM1_ESM.docx]

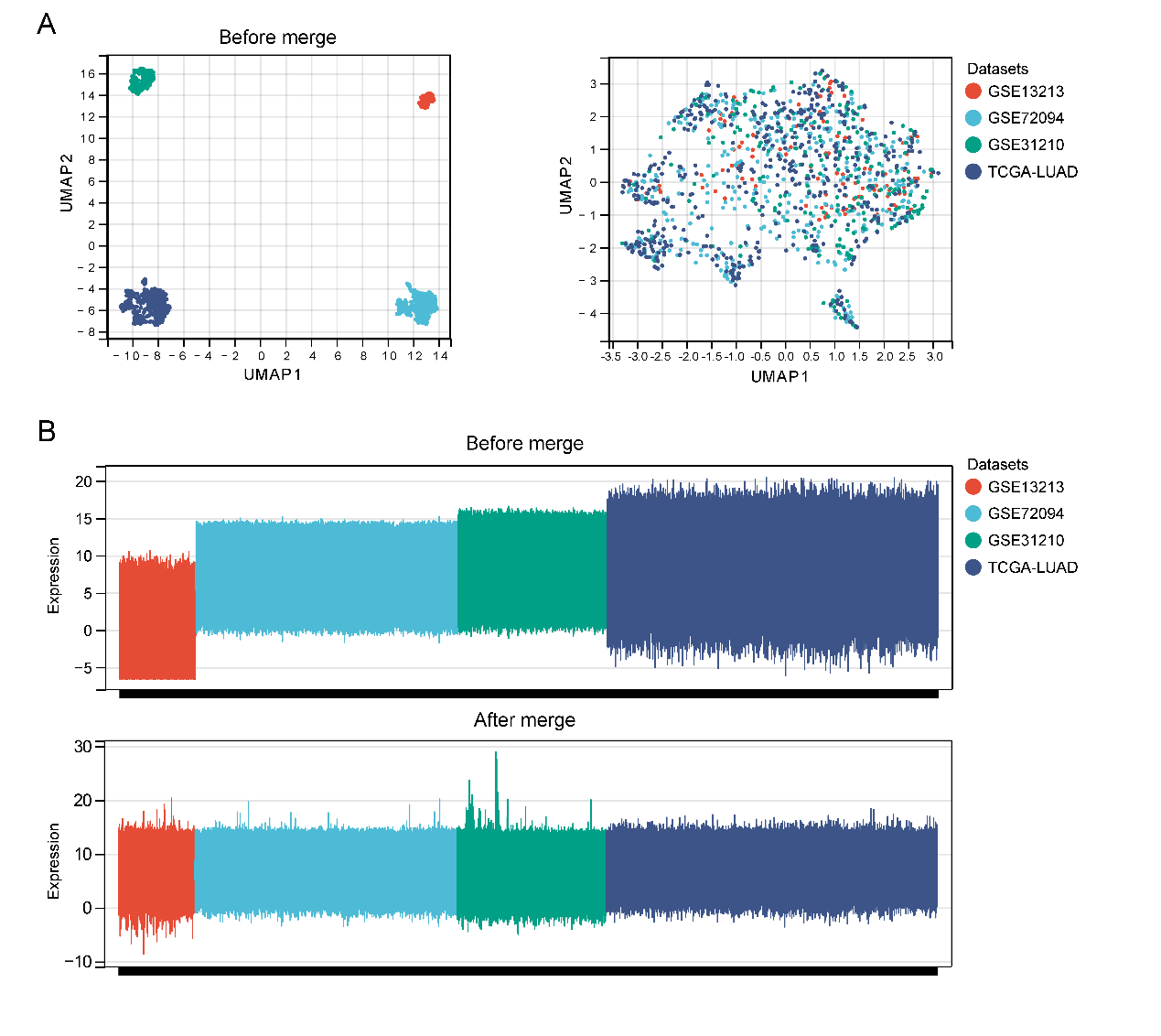


**Fig. S1** Pre-processing of LUAD number samples (A) Visualization of clustering results of LUAD samples before and after de-batching using UMAP plots (B) Box plots of expression values of LUAD samples before and after de-batching.


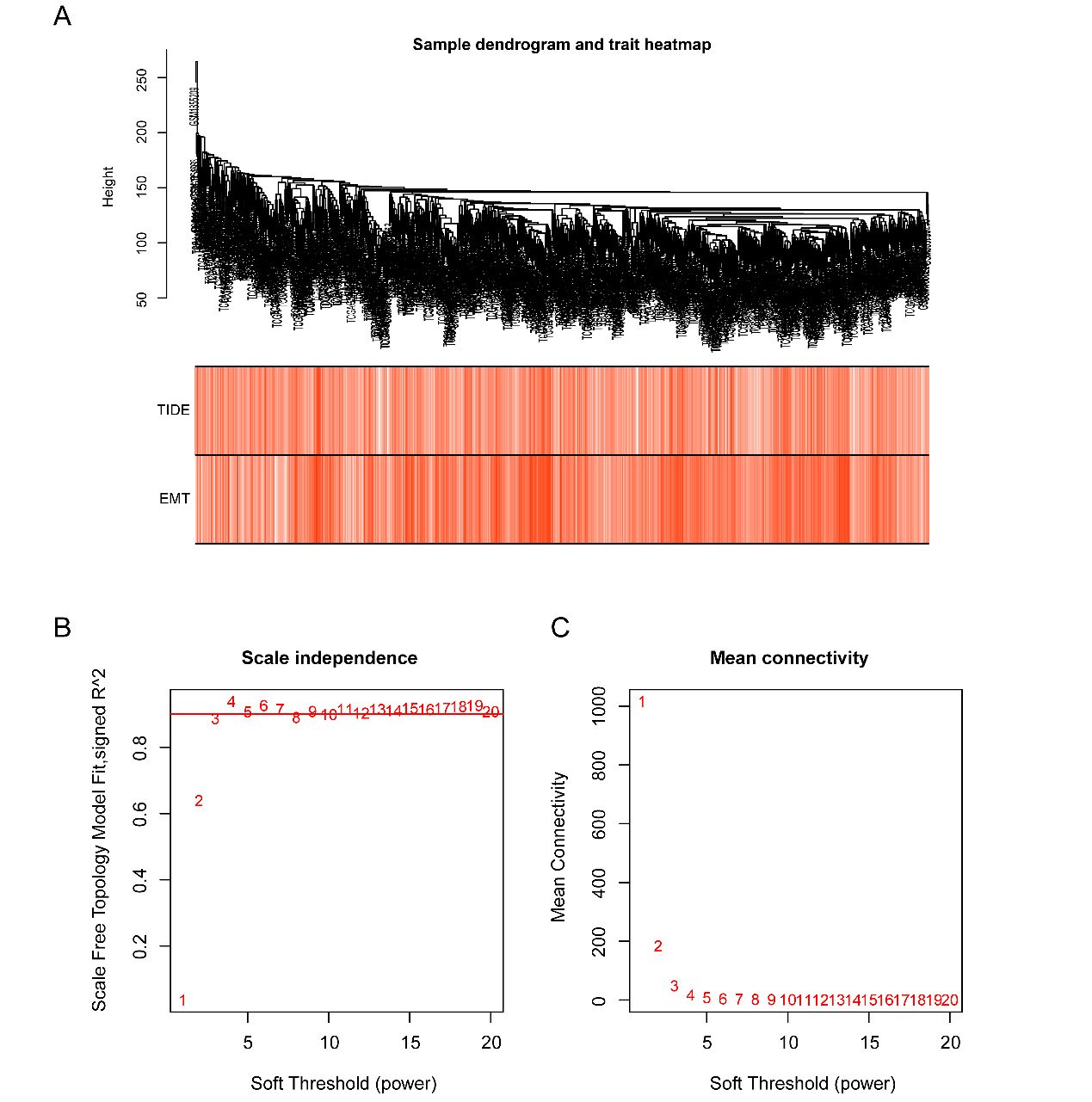


**Fig. S2 Gene co-expression network construction.** （A） Sample dendrogram and trait indicator. (B) Analyze the scale-free fit index of the 1-20 soft threshold power (β). (C) Analyze the average connectivity of 1-20 soft threshold power.


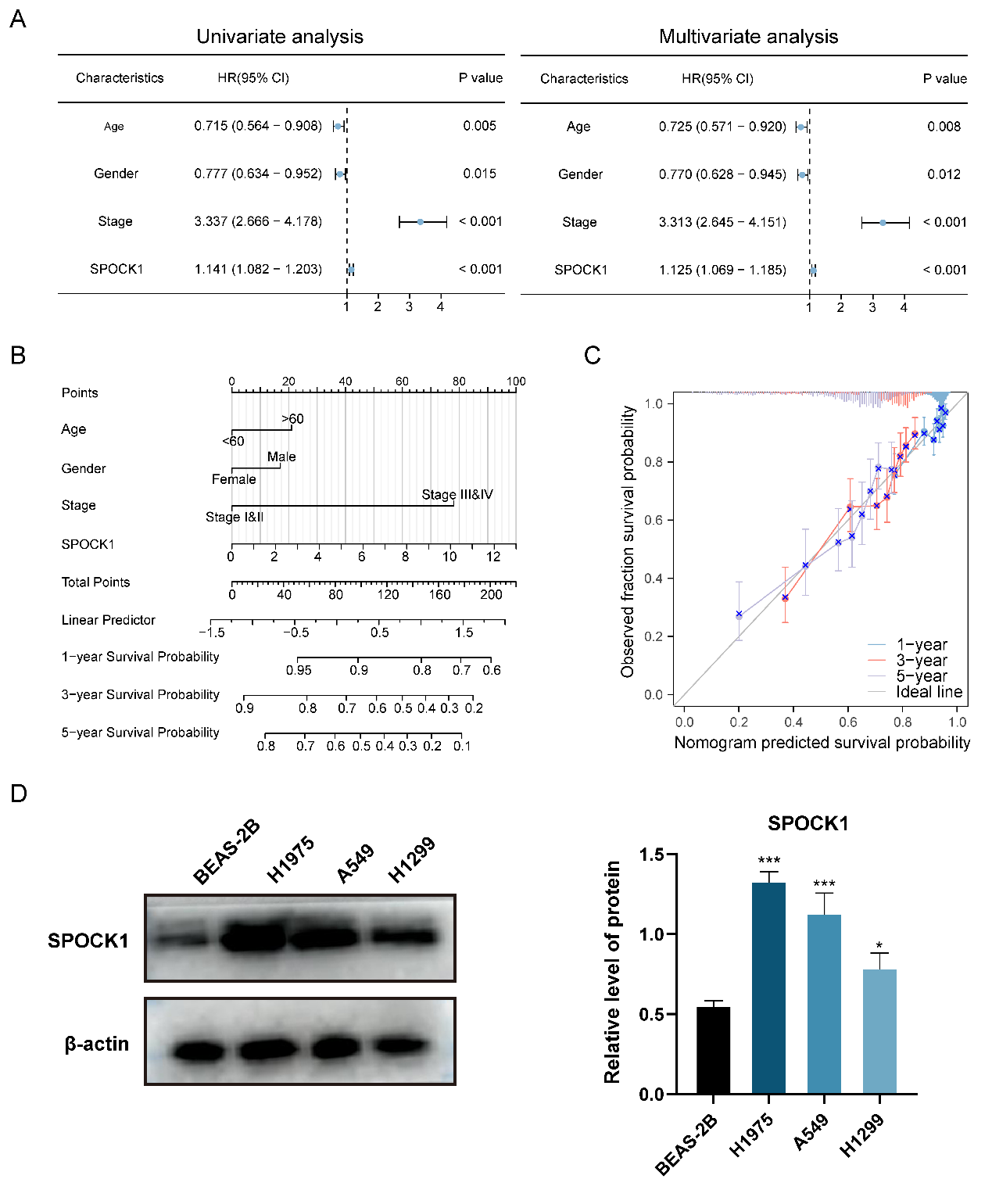


**Fig. S3 The prognostic value of the SPOCK1 in the combined cohort.** （A） Univariate and multivariate Cox regression analyses of the association between clinical features and OS of patients. (B) Construction of a nomogram for survival prediction based on SPOCK1. (C) The calibration curve for the nomogram model. Three colored lines (purple, red, and black) represent the performance of the nomogram. A closer fit to the diagonal gray line indicates a better estimation. (D) The western blot of SPOCK1 in normal lung cell lines and lung cancer cell lines


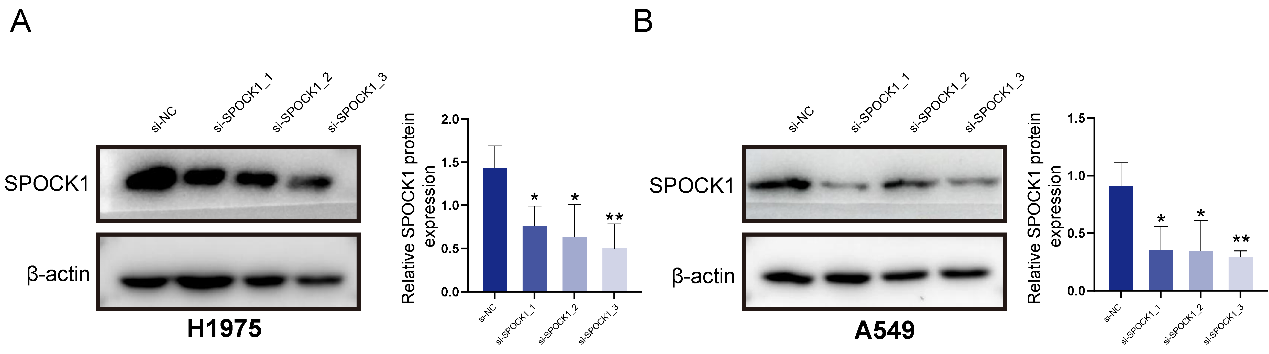


**Fig. S4 Knockdown of SPOCK1 in cell lines.** Western blotting assay after knockdown of SPOCK1 in （A）H1975 and （B）A549.


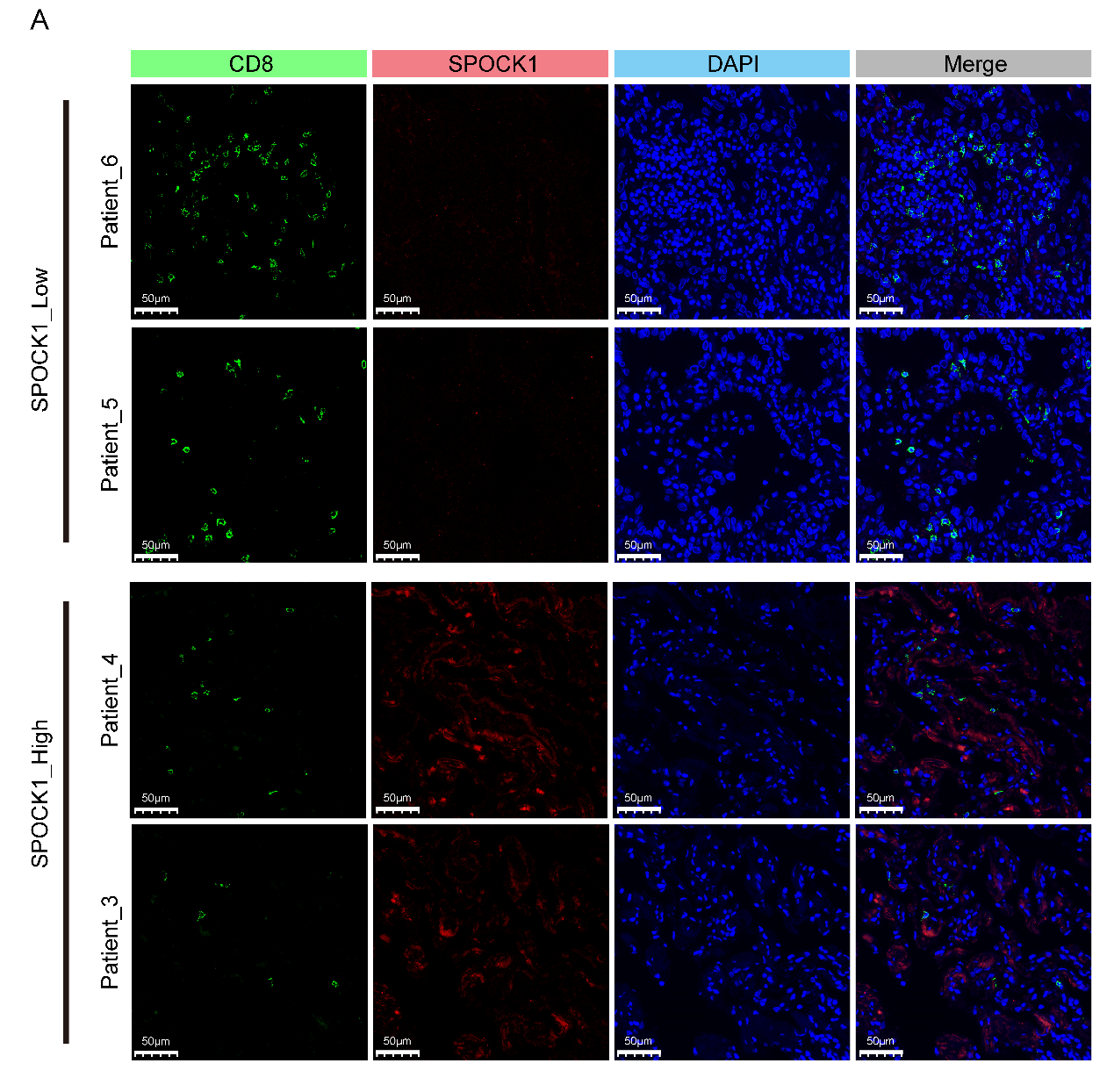


**Fig. S5 Immunofluorescence assay for SPOCK1 and CD8 in LUAD samples.**


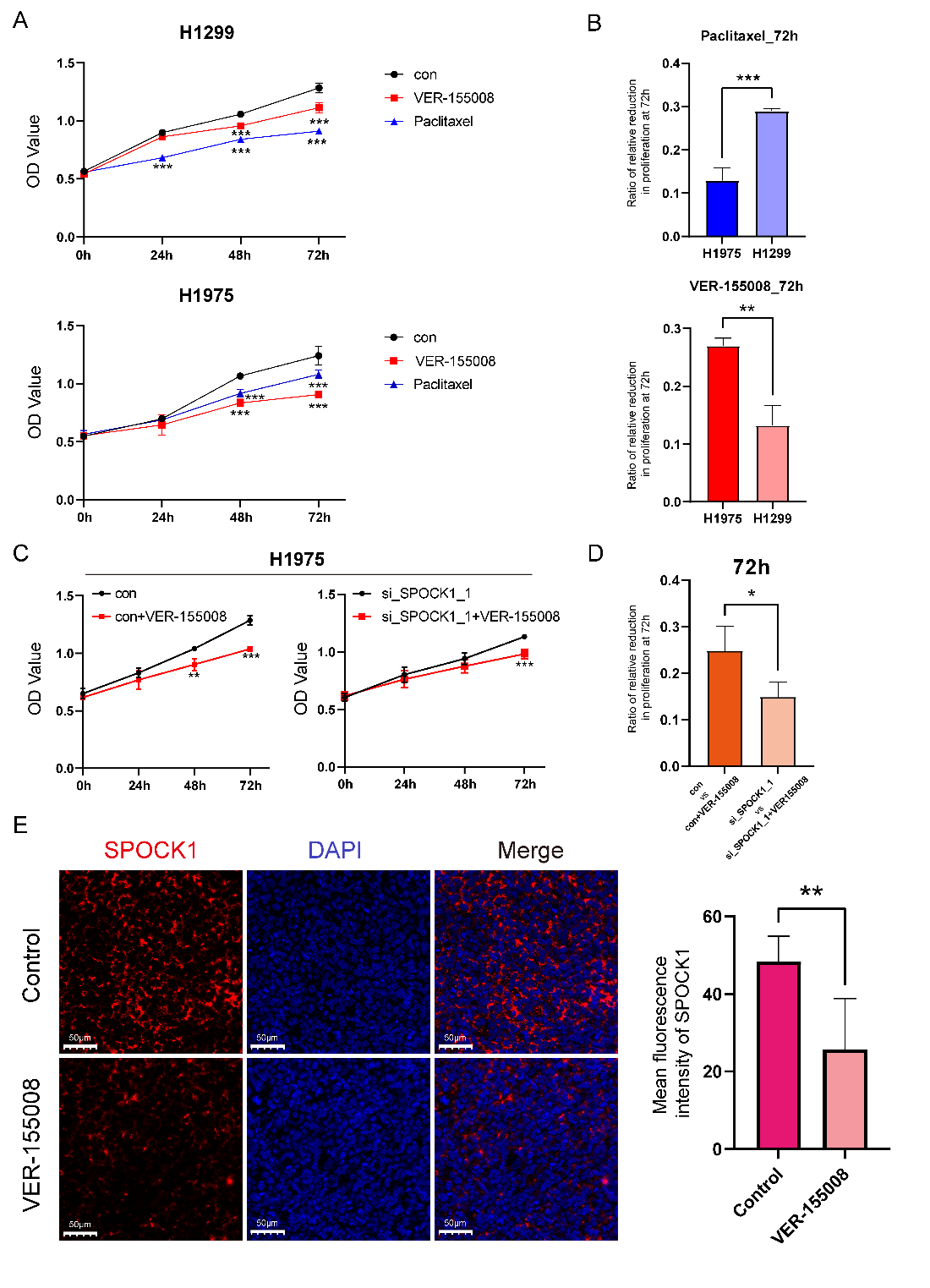


**Fig. S6 Validation of the candidate drug for SPOCK1.** (A) The proliferation of control, Paclitaxel((2μM)) or VER-155008(10μM) treated lung cancer cells was measured by MTS assay at the indicated time points. (B) The difference in the reduction of proliferation for Paclitaxel((2μM)) or VER-155008(10μM) treated lung cancer cells at 72h, Ratio of relative reduction in proliferation =（OD Value（con）- OD Value（VER-155008 or Paclitaxel））/OD Value（con）. (C) The proliferation of H1975 cells treated in the control group, VER-155008 group, si_SPOCK1_1 group, and si_SPOCK1_1 + VER-155008 group was detected by the MTS method at the indicated time points. (D) The difference in proliferation reduction between control+VER-155008 and si_SPOCK1_1+VER-155008 groups, after 72 hours of treatment of the H1975 cell line. (E) Immunofluorescence assay for SPOCK1 in control (n=6). or VER-155008 treated group (n=6).


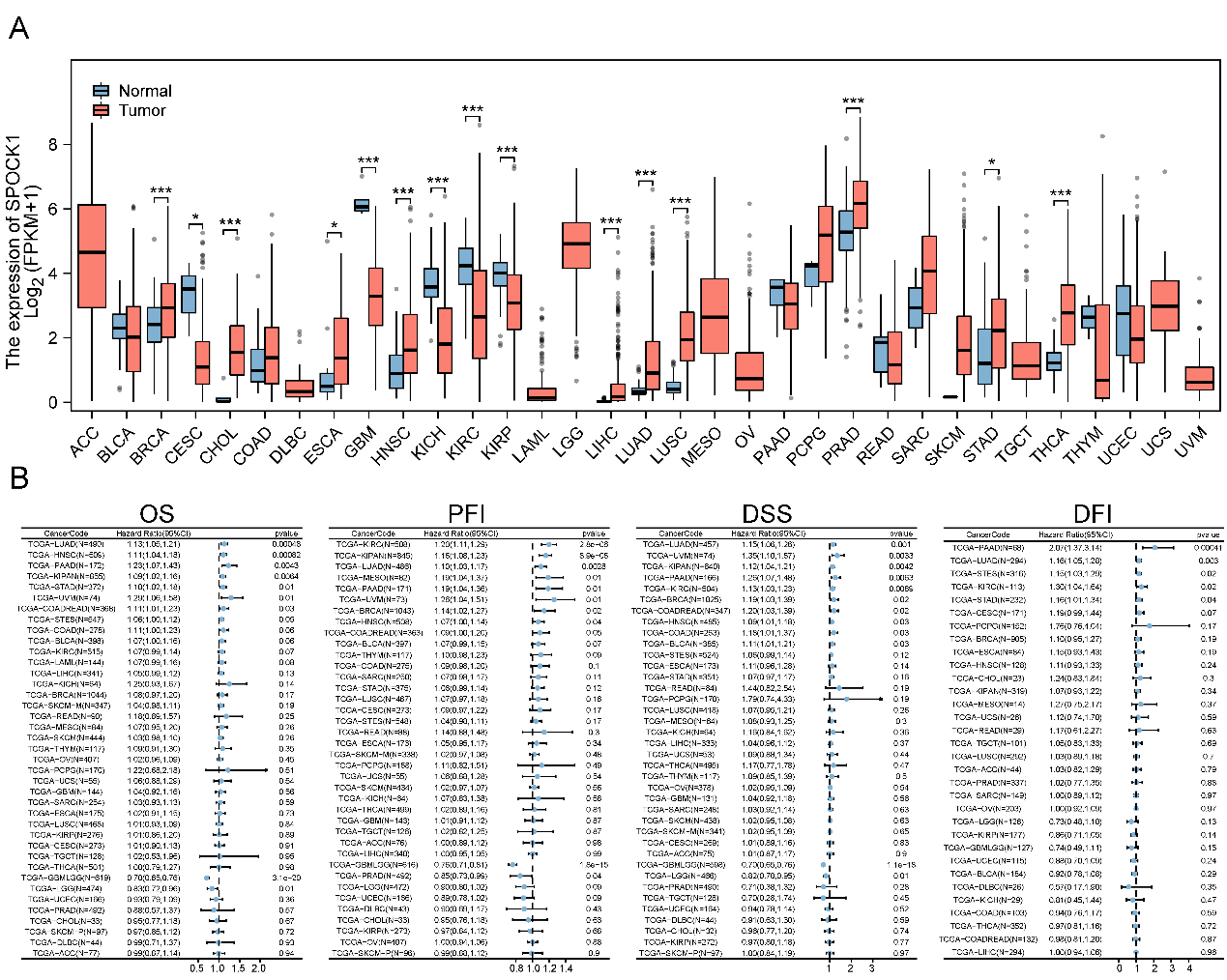


**Fig. S7 Pan-cancer study of SPOCK1.**（A）The mRNA expression of SPOCK1 between tumor and normal tissues was assessed from the TCGA database. （B）Univariate Cox regression analyses estimating prognostic value (OS/PFI/DSS/DFI) of SPOCK1 in pan-cancers from the TCGA database. （***p < 0.001; **p < 0.01; *p < 0.05; ns. no significance.）.
